# Changes in serum creatinine during and after pregnancy in female patients with or without chronic kidney disease: an observational study in UK primary care data

**DOI:** 10.1007/s40620-025-02208-6

**Published:** 2025-02-26

**Authors:** Carole A. Marxer, Julie M. Paik, Min Zhuo, Rishi J. Desai, Katrina Wilcox Hagberg, Susan S. Jick, Christoph R. Meier, Julia Spoendlin

**Affiliations:** 1 Hospital Pharmacy, University Hospital Basel, Basel, Switzerland; 2 Basel Pharmacoepidemiology Unit, Division of Clinical Pharmacy and Epidemiology, Department of Pharmaceutical Sciences, University of Basel, Basel, Switzerland; 3 Division of Pharmacoepidemiology and Pharmacoeconomics, Department of Medicine, Brigham and Women’s Hospital, Harvard Medical School, Boston, MA USA; 4 Division of Renal (Kidney) Medicine, Department of Medicine, Brigham and Women’s Hospital, Harvard Medical School, Boston, MA USA; 5 Division of Nephrology, Department of Medicine, Beth Israel Deaconess Medical Center, Harvard Medical School, Boston, MA USA; 6 Boston Collaborative Drug Surveillance Program, Lexington, MA USA; 7 Boston University School of Public Health, Boston, MA USA

**Keywords:** Obstetric nephrology, Renal filtration, Epidemiology, Clinical Practice Research Datalink (CPRD), Primary care

Renal filtration is increased throughout pregnancy, with peak filtration in the second trimester. This gestational hyperfiltration results in a continuous decrease in serum creatinine (SCr) levels in the first trimester, which plateaus in the second trimester, and gradually returns to baseline levels after delivery [1, 2].

Studies describing renal filtration in pregnant patients with severe chronic kidney disease (CKD) who were treated in tertiary care reported diminished hyperfiltration during pregnancy (Supplement-1.1.) [3, 4] However, evidence on renal filtration during pregnancy in patients with mild CKD who are seen in general practice is limited.

We aimed to describe changes in renal filtration during pregnancy and until one year afterwards within subgroups of women with different baseline estimated glomerular filtration rate (eGFR) measurements with a focus on women with mildly reduced eGFR.

We conducted a descriptive study using the United Kingdom (UK) primary care-based Clinical Practice Research Datalink (CPRD) GOLD and linked inpatient Hospital Episode Statistics (HES-APC) data. We included pregnancies from 2000–2019 among females aged 18–55 years at delivery with continuous enrollment from one year before until one year after pregnancy (observation period). We required ≥ 1 SCr measurement at baseline (one year before pregnancy) or during trimester 1, and ≥ 1 SCr measurement in trimesters 2/3 or in the postpartum period.

The outcome of interest was glomerular filtration as measured by recorded SCr values (CKD-EPI-2009 formula [5]). We categorized pregnancies by the median eGFR [ml/min/1.73m^2^] at baseline/trimester 1 based on the *Kidney Disease: Improving Global Outcomes* (KDIGO) G-categories: normal/high eGFR ≥ 90 (G1), mildly decreased eGFR = 60–89 (G2), or mildly to severely decreased eGFR = 15–59 (G3/G4, combined due to small sample size). We divided G2 into G2_high_ (eGFR = 75–89) and G2_low_ (eGFR = 60–74) because large sample size permitted in-depth analysis.

We present baseline characteristics and materno-fetal outcomes by eGFR category. We quantified median aggregate SCr levels (interquartile range, IQR) within two-week periods during pregnancies categorized as G1/G2_high_/G2_low_, and ten-week periods for G3/G4 pregnancies (smaller sample size) to describe changes in renal filtration over time. Further methods: Supplement-2.1.-2.7.

Of 14,401 pregnancies (13,791 females), 12,028 (83.5%) were categorized at baseline/trimester 1 as having normal renal filtration (G1), 1932 (13.3%) as G2_high_ mildly reduced filtration, 388 (2.7%) as G2_low_ mildly reduced filtration, and 53 (0.4%) as G3/G4. Pre-existing diabetes, hypertension, and/or BMI ≥ 25 kg/m^2^ were recorded in 51.3% of pregnancies categorized as G1, 50.6% in G2_high_, 54.4% in G2_low_, and 66.0% in G3/G4. A diagnosis for any renal disease was recorded in 2.3% and 5.2% of pregnancies categorized as G2_high_ and G2_low_, with 0.4% and 0.5% having a code for autoimmune kidney disease (56.6% and 3.8% for G3/G4, Table 1).

**Table 1:** Baseline characteristics and materno-fetal outcomes overall and by presence and severity of reduced baseline eGFR

	Overall	G1: Normal or high eGFR ≥ 90 ml/min/1.73 m^2^	G2_high_: Mildly decreased eGFR = 75–89 ml/min/1.73 m^2^	G2_low_: Mildly decreased eGFR = 60–74 ml/min/1.73 m^2^	G3/G4: Mildly to severely decreased eGFR = 15–59 ml/min/1.73 m^2^
Number of pregnancies	14,401 (100.0)	12,028 (83.5)	1932 (13.3)	388 (2.7)	53 (0.4)
Number of pregnant patients	13,791	11,539	1912	385	50
Baseline characteristics					
eGFR [ml/min/1.73m^2^] in baseline^a^ / trimester 1^b^, median [IQR]	110.72 [95.40,121.37]	114.58 [103.01,122.91]	83.96 [79.51,87.78]	70.56 [66.72,73.67]	49.61 [40.14,56.26]
SCr [mg/dL^c^] in baseline^a^/trimester 1^b^, median [IQR]	0.74 [0.66,0.84]	0.71 [0.63,0.78]	0.92 [0.88,0.96]	1.05 [1.01,1.1]	1.43 [1.26,1.72]
Age at delivery [years], median [IQR]	31.0 [27.0,35.0]	30.0 [26.0,34.0]	32.0 [29.0,36.0]	34.0 [31.0,38.0]	33.0 [30.0,37.0]
Calendar year of delivery					
2000–2004	982 (6.8)	690 (5.7)	228 (11.8)	53 (13.7)	11 (20.8)
2005–2009	3789 (26.3)	2762 (23.0)	823 (42.6)	182 (46.9)	22 (41.5)
2010–2014	6863 (47.7)	6062 (50.4)	668 (34.6)	118 (30.4)	15 (28.3)
2015–2019	2767 (19.2)	2514 (20.9)	213 (11.0)	35 (9.0)	5 (9.4)
Ethnicity					
Black	1080 (7.5)	1005 (8.4)	66 (3.4)	9 (2.3)	NR
White or other (except Black)	7952 (55.2)	6632 (55.1)	1093 (56.6)	201 (51.8)	26 (49.1)
Unknown	5369 (37.3)	4391 (36.5)	773 (40.0)	178 (45.9)	27 (50.9)
Current smoker^d^	1966 (13.7)	1634 (13.6)	278 (14.4)	45 (11.6)	9 (17.0)
Alcohol abuse^e^	471 (3.3)	385 (3.2)	69 (3.6)	15 (3.9)	NR
Number of SCr measurements					
Observation period^f^	39,956	33,386	5173	1150	247
Per pregnancy in baseline^a^ / trimester 1^b^, median [IQR]	1 [1, 2]	1 [1, 2]	1 [1, 2]	1 [1, 2]	2 [1, 2]
Per pregnancy in observation period^f^, median [IQR]	2 [2, 3]	2 [2, 3]	2 [2, 3]	2 [2, 3]	4 [2, 3]
≥ 3 per pregnancy	5639	4749	703	155	32
≥ 4 per pregnancy	2370	1984	278	81	27
≥ 5 per pregnancy	1116	930	116	47	23
≥ 6 per pregnancy	610	495	78	22	15
BMI [kg/m^2^]^g^					
Underweight (< 18.5)	487 (3.4)	438 (3.6)	40 (2.1)	9 (2.3)	NR
Normal weight (18.5 to < 24)	5153 (35.8)	4294 (35.7)	704 (36.4)	135 (34.8)	20 (37.7)
Overweight (25 to < 30)	2943 (20.4)	2482 (20.6)	370 (19.2)	79 (20.4)	12 (22.6)
Obesity (≥ 30)	2741 (19.0)	2277 (18.9)	371 (19.2)	86 (22.2)	7 (13.2)
Unknown	3077 (21.4)	2537 (21.1)	447 (23.1)	79 (20.4)	14 (26.4)
Pre-existing diabetes mellitus (DM)^h, i^	1467 (10.2)	1278 (10.6)	154 (8.0)	24 (6.2)	11 (20.8)
Pre-existing hypertension (HT)^h, j^	2448 (17.0)	1967 (16.4)	368 (19.0)	89 (22.9)	24 (45.3)
DM^h^ and/or overweight/obesity^g^	6239 (43.3)	5226 (43.4)	815 (42.2)	174 (44.8)	24 (45.3)
DM^h, i^ and/or HT^h, j^ and/or overweight/obesity^g^	7392 (51.3)	6169 (51.3)	977 (50.6)	211 (54.4)	35 (66.0)
Further potential causes of CKD					
Kidney disease^k^					
Overall	95 (0.7)	0 (0.0)	45 (2.3)	20 (5.2)	30 (56.6)
Autoimmune^l^	11 (0.1)	0 (0.0)	7 (0.4)	NR	NR
Non-immune-mediated^m^	23 (0.2)	0 (0.0)	7 (0.4)	6 (1.5)	10 (18.9)
Unspecified	61 (0.4)	0 (0.0)	31 (1.6)	12 (3.1)	18 (34.0)
Other autoimmune disease (exclusive autoimmune kidney disease)^k, n^	293 (2.0)	234 (1.9)	46 (2.4)	11 (2.8)	NR
Other potential cause^k, o^	89 (0.6)	75 (0.6)	11 (0.6)	NR	NR
Materno-fetal outcomes					
Gestational age at birth [weeks]^p^					
Term delivery (≥ 37 weeks)	12,917 (89.7)	10,780 (89.6)	1750 (90.6)	350 (90.2)	37 (69.8)
Preterm delivery (< 37 weeks)	1484 (10.3)	1248 (10.4)	182 (9.4)	38 (9.8)	16 (30.2)
Moderate to late preterm delivery (32 to 36 weeks)	974 (6.8)	798 (6.6)	133 (6.9)	32 (8.2)	11 (20.8)
Very preterm delivery (28 to 31 weeks)	176 (1.2)	148 (1.2)	21 (1.1)	NR	NR
Extremely preterm delivery (< 28 weeks)	334 (2.3)	302 (2.5)	28 (1.4)	NR	NR
Gestational age at birth [weeks], median [IQR]^p^	39.0 [38.0,40.0]	39.0 [38.0,40.0]	39.0 [38.0,40.0]	39.0 [38.0,40.0]	38.0 [36.0,40.0]
Birth weight [g]^q^					
Normal birth weight (≥ 2′500)	13,223 (91.8)	11,032 (91.7)	1797 (93.0)	353 (91.0)	41 (77.4)
Underweight (< 2′500)	907 (6.3)	754 (6.3)	111 (5.7)	30 (7.7)	12 (22.6)
Low birth weight (1′500–2′500)	773 (5.4)	645 (5.4)	93 (4.8)	27 (7.0)	8 (15.1)
Very low birth weight (1000–1′500)	86 (0.6)	68 (0.6)	13 (0.7)	NR	NR
Extremely low birth weight (< 1′000)	48 (0.3)	41 (0.3)	5 (0.3)	NR	2 (3.8)
Unknown	271 (1.9)	242 (2.0)	24 (1.2)	5 (1.3)	NR
Birth weight [g], mean (SD)^q, r^	3358.5 (588.5)	3359.2 (588.0)	3364.7 (577.6)	3370.4 (595.3)	2890.3 (830.4)
Number of previous pregnancies (live or stillborn), mean (SD)^s^	1.2 (1.4)	1.2 (1.4)	1.2 (1.4)	1.4 (1.6)	1.4 (1.9)
Method used to deliver the baby					
Spontaneous delivery	8037 (55.8)	6734 (56.0)	1070 (55.4)	208 (53.6)	25 (47.2)
Forceps delivery	872 (6.1)	746 (6.2)	112 (5.8)	13 (3.4)	NR
Vacuum extraction	845 (5.9)	700 (5.8)	122 (6.3)	20 (5.2)	NR
Breech delivery	64 (0.4)	58 (0.5)	5 (0.3)	NR	NR
Elective cesarean section	2046 (14.2)	1654 (13.8)	309 (16.0)	69 (17.8)	14 (26.4)
Emergency cesarean section	2228 (15.5)	1874 (15.6)	277 (14.3)	68 (17.5)	9 (17.0)
Unknown	309 (2.1)	262 (2.2)	37 (1.9)	9 (2.3)	NR

Values are the number (%) unless indicated otherwise

Abbreviations: eGFR, estimated glomerular filtration rate; IQR, interquartile range; SCr, serum creatinine; BMI, body mass index; DM, diabetes mellitus type 1 and 2; HT, hypertension; NR, not reported (because cell sizes < 5 patients); SD, standard deviation

^a^Baseline = (pregnancy start date -365 days) until pregnancy start date

^b^Trimester 1 = pregnancy start date until (pregnancy start date + 93days)

^c^Conversion factor for µmol/L: multiplication by 88.4

^d^Code for current smoker between start of baseline^a^ and delivery date

^e^READ code for ‘current’ and ≥ 14 alcohol units per week (average of units measured any time before end of trimester 1)

^f^Observation period = (pregnancy start date - 365 days) until (delivery date + 365 days)

^g^Last recording within three years before trimester 1 (classification according to Centers for Disease Control and Prevention. Defining Adult Overweight & Obesity. https://www.cdc.gov/obesity/adult/defining.html. Published 2021. Accessed April 4, 2021)

^h^Last recording any time before end of trimester 1^b^

^i^Diagnosis code and/or drug prescription and/or HbA1c level ≥ 6.5%

^j^Diagnosis code and/or drug prescription

^k^Last recording any time prior to the delivery date (excluded in G1 group)

^l^Chronic glomerulonephritis and/or vasculitis (small and medium vessel vasculitis, i.e. polyarteritis nodosa, microscopic polyangiitis, Kawasaki disease, Churg-Strauss disease, Wegener’s granulomatosis, arteritis not otherwise specified, Henoch-Schonlein purpura, hypersensitivity angiitis) and/or hemolytic uremic syndrome and/or other miscellaneous immune-mediated kidney disease

^m^Cystic kidney disease and/or other miscellaneous non-immune-mediated kidney disease

^n^Systemic lupus erythematosus and/or rheumatoid arthritis or other inflammatory polyarthropathies and/or Sjögren syndrome or sicca syndrome and/or dermatomyositis or polymyositis and/or systemic sclerosis and/or other connective tissue disease (incl. polymyalgia rheumatica, eosinophilia myalgia))

^o^Amyloidosis and/or gout and/or chronic liver disease

^p^Definition by the World Health Organization. Preterm birth. Published 2018. Accessed August 2, 2021. https://www.who.int/news-room/fact-sheets/detail/preterm-birth

^q^Definition by the World Health Organization. International statistical classification of diseases and related health problems: tenth revision, 2nd ed. World Health Organization. Published 2004. Accessed April 4, 2021. https://apps.who.int/iris/handle/10665/42980

^r^Missing in 271 pregnancies

^s^Missing in 3040 pregnancies

Preterm birth (< 37 gestational weeks) and low-birth weight of the infant (< 2500 g) were recorded in 9.4% and 5.7% of pregnancies categorized as G2_high_ and 9.8% and 7.7% in G2_low_ pregnancies. Among G3/G4 pregnancies, 30.2% were preterm and 22.6% were underweight births (Table 1).

The pattern of changes in SCr levels was similar in pregnancies categorized as G1 and G2_high_ (at higher baseline SCr levels for G2_high_, Fig. 1/Table 1). Median SCr levels (mg/dl [µmol/L]) during baseline/trimester 1 were 0.71 [62.76] (IQR = 0.63–0.78 [55.69–68.95]) for G1, and 0.92 [81.33] (IQR = 0.88–0.96 [77.79–84.86]) for G2_high_. In both groups, SCr levels continuously decreased from baseline until week 14/15 (G1: -0.16 [-14.14], G2_high_: -0.25 [-22.1]), after which SCr levels remained stable until week 30/31. Subsequently, SCr levels increased steadily and returned to baseline around week 5/6 postpartum.

**Fig. 1:**
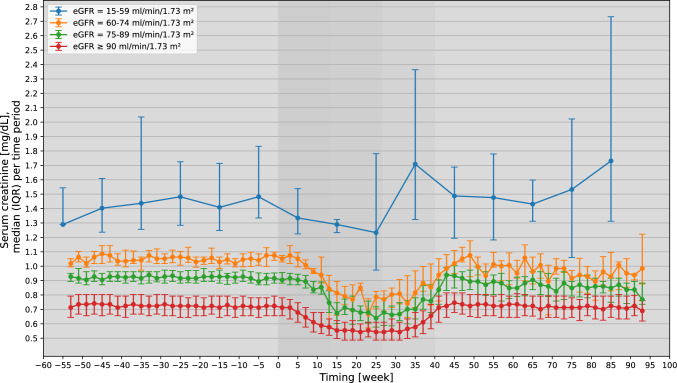
Median SCr levels (IQR) [mg/dL*] during baseline (before week 0), pregnancy (weeks 0–40), and postpartum period (after week 40). Changes in SCr levels are presented as two-week period medians among pregnancies categorized as G1 (eGFR ≥ 90 ml/min/1.73 m^2^), G2_high_ (eGFR = 75–89 ml/min/1.73 m^2^), or G2_low_ (eGFR = 60–74 ml/min/1.73 m^2^) at baseline, and as ten-week period medians among pregnancies categorized as G3/G4 (eGFR = 15–59 ml/min/1.73 m^2^). Median SCr levels are placed in the center of each time period. *Conversion factor for µmol/L: multiplication by 88.4

Among G2_low_ pregnancies, median SCr levels during baseline/trimester 1 were 1.05 [92.82] (IQR = 1.01–1.10 [89.28–97.24]) and decreased until week 18/19 (-0.28 [-24.75]), followed by a plateau until week 34/35. Afterwards, SCr increased to above-baseline values in week 9/10 postpartum (1.07 [94.59], IQR = 0.93–1.18 [82.21–104.31]), after which values returned to baseline.

In G3/G4, the baseline/trimester 1 median SCr level was 1.43 [126.41] (IQR = 1.26–1.72 [111.38–152.05]). After a decrease in recorded SCr levels in trimester 1/2, they surpassed baseline levels by week 30/40 of pregnancy (median = 1.71 [151.16], IQR = 1.32–2.36 [116.69–208.62]) and decreased again thereafter. However, sample size for G3/G4 was small. Further results: Supplement-3.1.-3.4.

Our study describes changes in renal filtration in a large cohort of pregnancies (N = 2320) in patients with mildly decreased eGFR seen in routine primary care. Our results suggest that similar to a prior study [1], mildly reduced renal filtration (eGFR = 75–89, G2_high_) at baseline/trimester 1 does not impair physiological changes in filtration during or after pregnancy.

In pregnancies with eGFR = 60–74 (G2_low_), SCr levels increased more slowly (vs. patients with normal filtration) during trimester 3 and early postpartum (until 9–10 weeks postpartum), which might indicate increased stress on the kidneys and a slower return to baseline filtration. We cannot rule out bias due to selective SCr measurements in pregnancies with complications, but these results warrant further research.

SCr patterns among patients with moderately-severely reduced renal filtration (G3/G4) at baseline/trimester 1 must be interpreted cautiously due to small sample size and potential selection bias due to the referral to secondary care of more severe cases (further discussion: Supplement-4.3.).

Preterm births and births of underweight infants were comparable between all patients with mildly reduced renal filtration (G2_high_, G2_low_) and patients with normal renal filtration, which suggests that mildly reduced renal filtration does not independently increase the risk for these neonatal outcomes. However, both outcomes were more prevalent in patients with normal or mildly reduced renal filtration, when compared to the UK general population (preterm birth: 9.8% vs. 7.6%; underweight: 7.7% vs. 7.0%) [6]. This is likely mediated by the higher prevalence of metabolic risk factors among patients with SCr recordings, as NICE guidelines [7] recommend to monitor eGFR in the presence of diabetes, hypertension, or obesity. Such monitoring is financially incentivized in UK general practitioner (GP) practice (Supplement-4.1.). Substantially increased proportions for preterm birth and underweight infant were observed in patients with renal filtration G3/G4 (30.2% and 22.6%), which is consistent with prior studies [3, 4].

The main limitation of this study is the under-recording of CKD and albuminuria in primary care in the UK [8].Thus, eGFR was the closest approximation of kidney function (for further limitations see Supplement-4.5.).

In conclusion, adaptation of renal filtration was not compromised in pregnancies with eGFR = 75–89. The prolonged increase in SCr levels in pregnancies with eGFR < 75 needs further investigation before speculating on the clinical implications of this finding.

## Supplementary Information

Below is the link to the electronic supplementary material.

Supplementary file1 (DOCX 675 KB)

## Data Availability

Owing to official regulations by the data provider, the sharing of analytical data is strictly prohibited. Any analytical data has to be stored on a pre-specified and agreed-on server with strict access control. Data can be requested through an official data request submitted to the Clinical Practice Research Datalink (CPRD). Data for this study were derived from CPRD GOLD primary care data obtained under license from the UK Medicines and Healthcare products Regulatory Agency. The data are provided by patients and collected by the NHS as part of their care and support. The interpretation and conclusions contained in this study are those of the authors alone. HES Maternity data Copyright © (2020) were re-used with the permission of The Health & Social Care Information Centre. All rights reserved. This study was approved by the Independent Scientific Advisory Committee (ISAC) for Medicines and Healthcare products Regulatory Agency database research (protocol no: 20_004R, made available to editors and reviewers).
