# Supplementary material for: Changes in serum creatinine during and after pregnancy in female patients with or without chronic kidney disease: an observational study in UK primary care data
Source: J Nephrol. 2025 Feb 26;38(5):1509–15. doi: 10.1007/s40620-025-02208-6 (PMC12289759; doi:10.1007/s40620-025-02208-6)
Supplement: Supplementary file 1 — Supplementary file1 (DOCX 675 KB) [file 40620_2025_2208_MOESM1_ESM.docx]

**SUPPLEMENTARY MATERIAL**

Title: Changes in Serum Creatinine during and after Pregnancy in Female Patients with or without Chronic Kidney Disease: An Observational Study in UK Primary Care Data

**Table of contents**

[**1.** **Further background** 2](#_Toc183164846)

[1.1. Previous research 2](#_Toc183164847)

[**2.** **Further methods** 3](#_Toc183164848)

[2.1. Data source 3](#_Toc183164849)

[2.2. Study population 3](#_Toc183164850)

[Final (linked) study population 3](#_Toc183164851)

[Flow chart 4](#_Toc183164852)

[2.3. CPRD GOLD population 4](#_Toc183164853)

[2.4. Extraction of serum creatinine 11](#_Toc183164854)

[Overview 11](#_Toc183164855)

[Read codes used to identify SCr in CPRD 11](#_Toc183164856)

[2.5. Categorization of pregnancies into KDIGO G-categories 11](#_Toc183164857)

[2.6. Covariables 12](#_Toc183164858)

[Overview 12](#_Toc183164859)

[2.7. Statistical analyses 12](#_Toc183164860)

[**3.** **Further results** 13](#_Toc183164861)

[3.1. Numeric information for Figure 1 (changes in SCr levels over time) 13](#_Toc183164862)

[3.2. Baseline characteristics of CPRD GOLD population 19](#_Toc183164863)

[3.3. Changes in SCr levels in CPRD GOLD population 22](#_Toc183164864)

[3.4. Numeric information of Figure S2 (changes in SCr over time) in CPRD GOLD population (not used for final analyses) 24](#_Toc183164865)

[**4.** **Further discussion** 30](#_Toc183164866)

[4.1. CPRD GOLD as unique data source for our research question 30](#_Toc183164867)

[4.2. Baseline characteristics 30](#_Toc183164868)

[4.3. Pregnancies categorized as G3/G4: Changes in SCr levels and representativeness of the G3/G4 category 30](#_Toc183164869)

[4.4. Materno-fetal outcomes 31](#_Toc183164870)

[4.5. Limitations 31](#_Toc183164871)

[**5.** **References (section 2, 3, 4)** 32](#_Toc183164872)

# **Further background**

## Previous research

- Davison JM, Dunlop W, Ezimokhai M. 24-hour creatinine clearance during the third trimester of normal pregnancy. BJOG. 1980;87:106–109.
- Davison JM, Noble MCB. Serial changes in 24 hour creatinine clearance during normal menstrual cycles and the first trimester of pregnancy. BJOG. 1981;88:10–17.
- Roberts M, Lindheimer MD, Davison JM. Altered glomerular permselectivity to neutral dextrans and heteroporous membrane modeling in human pregnancy. Am J Physiol. 1996;270:F338–F343.
- Chapman AB, Abraham WT, Zamudio S, et al. Temporal relationships between hormonal and hemodynamic changes in early human pregnancy. Kidney Int. 1998;54: 2056–2063.
- Katz AI, Davison JM, Hayslett JP, Singson E, Lindheimer MD. Pregnancy in women with kidney disease. Kidney Int. 1980;18(2):192-206. doi:10.1038/ki.1980.128
- Surian M, Imbasciati E, Cosci P, et al. Glomerular Disease and Pregnancy: A study of 123 pregnancies in patients with primary and secondary glomerular diseases. Nephron. 1984;(36):101-105. doi:10.1159/000183126
- Abe S, Amagasaki Y, Konishi K, Kato E, Sakaguchi H, Iyori S. The influence of antecedent renal disease on pregnancy. Am J Obstet Gynecol. 1985;153(5):508-514. doi:10.1016/0002-9378(85)90463-6
- Packham DK, North RA, Fairley KF, Kloss M, Whitworth JA, Kincaid-Smith P. Primary Glomerulonephritis and Pregnancy. Q J Med. 1989;71(3):537-553. doi:10.1093/oxfordjournals.qjmed.a068348
- Hou SH, Grossman SD, Madias NE. Pregnancy in women with renal disease and moderate renal insufficiency. Am J Med. 1985;78(2):185-194. doi:10.1016/0002-9343(85)90425-5
- Barcelo P, Lopez-Lillo J, Cabero L, Del Rio G. Successful pregnancy in primary glomerular disease. Kidney Int. 1986;30(6):914-919. doi:10.1038/ki.1986.272
- Jungers P, Houillier P, Chauveau D, et al. Pregnancy in women with reflux nephropathy. Kidney Int. 1996;50(2):593-599. doi:10.1038/ki.1996.354
- Jungers P, Chauveau D, Choukroun G, et al. Pregnancy in women with impaired renal function. Clin Nephrol. 1997;47(5):281-288.
- Cunningham FG, Cox SM, Harstad TW, Mason RA, Pritchard JA. Chronic renal disease and pregnancy outcome. Am J Obstet Gynecol. 1990;163(2):453-459. doi:10.1016/0002-9378(90)91175-C

# **Further methods**

## Data source

The Clinical Practice Research Datalink (CPRD) GOLD is a longitudinal database containing electronic primary care health records of 7% of the UK population.^1^ Recorded information includes demographics, prescriptions, diagnoses, life-style factors, and referrals to and diagnoses from secondary care. Results of laboratory tests ordered by the general practitioner (GP) are directly recorded via electronic links to laboratories.^1^ We linked CPRD GOLD data to Hospital Episode Statistics Admitted Patient Care (HES-APC) data (Maternity data), which covers all hospital admissions for patients in England covered by the National Health Service (58% of GPs have HES linkage), and includes information on delivery hospitalizations (gestational age at birth, birth weight etc.).^1,2^

## Study population

### Final (linked) study population

Among all females (aged 18-55 years) in CPRD GOLD with linked HES-APC data, we selected pregnancies based on diagnostic Read codes indicating delivery, post-natal and pregnancy codes, between 1/1/2000 and 12/31/2019. Delivery codes recorded <134 days apart were considered part of the same pregnancy, because a new delivery is physiologically not possible within 134 days after delivery, and delayed recording of delivery codes is possible in CPRD GOLD. 1.03% of pregnancies were identified based on post-natal and pregnancy codes.^3^ Each patient could contribute multiple eligible pregnancies to the study population.

We excluded pregnancies that resulted in multiple births based on Read codes because physiological changes of SCr may be different in those pregnancies, and sample size was too small for sensitivity analyses. To ensure that there was detailed information on gestational age, we restricted the population to pregnancies where the exact gestational age at birth (GAB) was recorded in HES-APC maternity data. The delivery date and the GAB were extracted from HES-APC maternity data and the last menstrual period (LMP) was calculated based on this information.

We required patients to have continuous enrollment in CPRD GOLD from one year prior to LMP to one year after delivery date (observation period). The observation period was divided into a one-year baseline period before LMP, pregnancy, and a one-year postpartum period. All pregnancies were required to have >=1 recorded SCr measurement in the baseline/trimester 1 and >=1 recorded SCr measurement in trimester 2/3 or postpartum. We excluded pregnancies with a median eGFR<15ml/min/1.73m^2^ (kidney failure) during baseline/trimester 1, because changes in SCr levels in those patients are not physiological (i.e., influenced by dialysis or kidney transplantation). Finally, we excluded pregnancies if there was a Read code for cancer, human immunodeficiency virus (HIV), substance abuse, hemodialysis, or kidney transplantation at any time before the end of the observation period, or where a Read code for acute kidney injury was recorded during the observation period. Patients with a Read code for a prior delivery during baseline were also excluded. We censored follow-up in the postpartum period for pregnancies at the start date of a new pregnancy.

A pregnancy population based on CPRD GOLD data only was established (CPRD GOLD population, second last row in flow chart below) but was not used for analyses due to insufficient recording of GAB. Further information about the CPRD GOLD population is provided below (estimation of the pregnancy start date and validation of the estimated pregnancy start date in the CPRD GOLD population).

### Flow chart

**
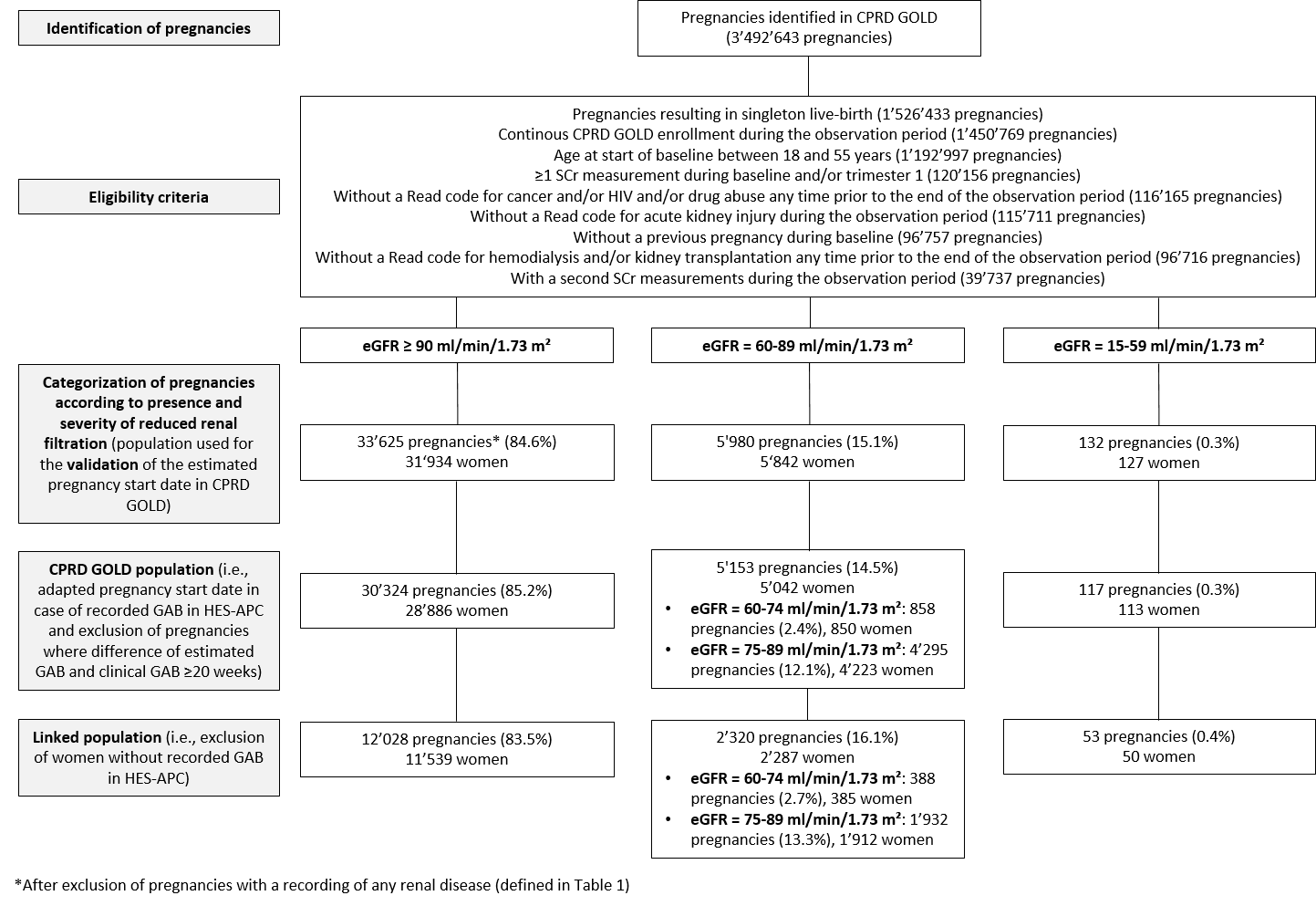
**

Abbreviations: CPRD = Clinical Practice Research Datalink, SCr = serum creatinine, HIV = human immunodeficiency virus, CKD = chronic kidney disease, LMP = last menstrual period, eGFR = estimated glomerular filtration rate, GAB = gestational age at birth, HES-APC = Hospital Episode Statistics Admitted Patient Care

## CPRD GOLD population

#### Estimation of the pregnancy start date

The algorithm to define the pregnancy start date was organized in a hierarchical manner and we used Read codes for the last menstrual period (LMP), estimated delivery date, and preterm delivery recorded in CPRD GOLD. In total, 32.6% of pregnancies had a recorded Read code for LMP within 310 days before delivery date indicating the pregnancy start date. In the absence of a LMP Read code, we estimated the pregnancy start date as 1) 280 days before a recorded Read code indicating the estimated date of delivery (if it coincided with the delivery date by ±90 days) (0.9%), 2) 245 days before delivery date in the presence of a recorded Read code (±90 days of delivery date) indicating preterm delivery (0.3%), and 3) 280 days before the delivery date if none of the above were applicable (66.2%).

#### Validation of the estimated pregnancy start date

Background

In the CPRD GOLD population, we validated the assignment of pregnancy start date, delivery date, and gestational age at birth (GAB) to assess potential misclassification of the observation period using linked CPRD GOLD and HES-APC data.

We based our final analyses on patients with HES-APC linkage and recorded GAB (linked population) because preterm deliveries were under-recorded in CPRD GOLD, and therefore the median difference between the estimated GAB in CPRD GOLD (delivery date – estimated pregnancy start date) and the actually recorded GAB in HES-APC data (external reference standard) was large among pregnancies with preterm deliveries in HES-APC data (<37 completed weeks of gestation, median difference=4 weeks). To create the linked (final) study population, we replaced the estimated pregnancy start date in CPRD GOLD with the LMP recorded in HES-APC data, excluding all patients for whom no recorded GAB from HES-APC data was available. We then further excluded pregnancies if 1) they no longer fulfilled the eligibility criteria due to changes in the observation period (n=4’071; where the required baseline/trimester 1 SCr measurement shifted to trimester 2 based on the LMP recorded in HES-APC), and 2) if the difference between the estimated and clinical GAB was ≥20 weeks (n=72; presumed recording errors).

Methods

We compared the estimated pregnancy start date in CPRD GOLD to the clinical GAB (external reference standard) recorded in the HES-APC maternity dataset among 14’565 pregnancies with recorded clinical GAB (i.e., 76.4%). The median difference (in weeks) between the estimated GAB in CPRD GOLD (delivery date – estimated LMP) and the recorded clinical GAB in HES-APC data was 0 overall and across all groups (IQR_overall_=[-1.0, 0.0], IQR_eGFR≥90_=[-1.0, 0.0], IQR_eGFR=60-89_=[-1.0, 0.0], IQR_eGFR=15-59_=[-2.0, 0.0]). However, only 0.9% of pregnancies in CPRD GOLD had a recorded code for preterm delivery (eGFR [ml/min/1.73 m^2^] ≥90 [KDIGO G1]: 0.9%, eGFR=60-89 [KDIGO G2]: 0.9%, eGFR=15-59 [KDIGO G3/G4]: 1.9%), whereas the proportion of preterm deliveries (GAB <37 completed weeks) in HES-APC was 10.3% (G1: 10.3%, G2: 9.5%, G3/G4: 30.3%) which is consistent with national statistics.^[[1]](#footnote-1)^ The median difference (in weeks) was larger among pregnancies classified as preterm delivery in HES-APC (G1: -4.0, IQR=[-12.0, 0.0], G2: -4.0, IQR=[-6.3, 0.0], G3/G4: -4.5, IQR=[-7.8, 0.0]).

Results

Table S1: Validation of the estimated pregnancy start date in CPRD GOLD overall, by presence and severity of reduced renal filtration, and separately for term and preterm deliveries

|  |  | **Term and preterm deliveries** | **Term deliveries**^[[2]](#footnote-2)^ | **Preterm deliveries**^[[3]](#footnote-3)^ |
| --- | --- | --- | --- | --- |
| **Overall:** | | | | |
| **Number of pregnancies (%)** | Total | 39737 | - | - |
|  | …with HES-APC linkage | 20477 | - | - |
|  | …with matching pregnancy in HES-APC | 19060 (48.0%) | 17525 | 1534 |
|  | ...with recorded clinical GAB | 14565 | 13031 | 1534 |
| **Clinical GAB, percentiles** | 0% (minimum)  1%  5%  10%  20%  25%  30%  40%  50% (median)  60%  70%  75%  80%  90%  95%  99%  100% (maximum) | 2.0  13.0  34.0  36.0  38.0  38.0  38.0  39.0  39.0  40.0  40.0  40.0  40.0  41.0  41.0  42.0  49.0 | 37.0  37.0  37.0  38.0  38.0  38.0  39.0  39.0  39.0  40.0  40.0  40.0  41.0  41.0  41.0  42.0  49.0 | 2.0  10.0  11.0  13.3  27.0  28.0  29.0  33.0  34.0  35.0  35.0  36.0  36.0  36.0  36.0  36.0  36.0 |
| **Clinical GAB – estimated GAB, percentiles** | 0% (min)  1%  5%  10%  20%  25%  30%  40%  50% (median)  60%  70%  75%  80%  90%  95%  99%  100% (max) | -41.0  -26.0  -5.0  -3.0  -2.0  -1.0  -1.0  0.0  0.0  0.0  0.0  0.0  1.0  1.0  2.0  6.0  40.0 | -14.0  -4.0  -3.0  -2.0  -1.0  -1.0  -1.0  0.0  0.0  0.0  0.0  0.0  1.0  1.0  2.0  6.0  40.0 | -41.0  -31.0  -29.0  -26.0  -12.0  -12.0  -9.0  -5.0  -4.0  -4.0  -1.0  0.0  0.0  0.0  1.0  5.0  19.0 |
| **Clinical GAB – estimated GAB, number of pregnancies (%)** | 0  negative  positive  1 or -1  2 or -2  3 or -3  4 or -4  ≥5 or ≤-5 | 5900 (40.51)  5549 (38.1)  3116 (21.39)  4698 (32.26)  1763 (12.1)  741 (5.09)  383 (2.63)  1080 (7.42) | 5571 (42.75)  4471 (34.31)  2989 (22.94)  4538 (34.82)  1717 (13.18)  710 (5.45)  161 (1.24)  334 (2.56) | 329 (21.45)  1078 (70.27)  127 (8.28)  160 (10.43)  46 (3.0)  31 (2.02)  222 (14.47)  746 (48.63) |
| **KDIGO G1 (eGFR ≥ 90 ml/min/1.73m^2^):** | | | | |
| **Number of pregnancies (%)** | Total | 33625 | - | - |
|  | …with HES-APC linkage | 16863 | - | - |
|  | …with matching pregnancy in HES-APC | 15723 | 14436 | 1286 |
|  | ...with recorded clinical GAB | 12158 | 10872 | 1286 |
| **Clinical GAB, percentiles** | 0% (minimum)  1%  5%  10%  20%  25%  30%  40%  50% (median)  60%  70%  75%  80%  90%  95%  99%  100% (maximum) | 2.0  12.0  34.0  36.0  38.0  38.0  38.0  39.0  39.0  40.0  40.0  40.0  40.0  41.0  41.0  42.0  49.0 | 37.0  37.0  37.0  38.0  38.0  38.0  39.0  39.0  39.0  40.0  40.0  40.0  41.0  41.0  41.0  42.0  49.0 | 2.0  10.0  11.0  13.0  26.0  28.0  28.0  32.0  34.0  35.0  35.0  36.0  36.0  36.0  36.0  36.0  36.0 |
| **Clinical GAB – estimated GAB, percentiles** | 0% (min)  1%  5%  10%  20%  25%  30%  40%  50% (median)  60%  70%  75%  80%  90%  95%  99%  100% (max) | -41.0  -27.0  -5.0  -3.0  -2.0  -1.0  -1.0  0.0  0.0  0.0  0.0  0.0  1.0  1.0  2.0  6.0  40.0 | -14.0  -4.0  -3.0  -2.0  -1.0  -1.0  -1.0  0.0  0.0  0.0  0.0  0.0  1.0  1.0  2.0  6.0  40.0 | -41.0  -31.0  -29.0  -26.0  -13.0  -12.0  -10.5  -6.0  -4.0  -4.0  -1.0  0.0  0.0  0.0  1.0  4.0  19.0 |
| **Clinical GAB – estimated GAB, number of pregnancies (%)** | 0  negative  positive  1 or -1  2 or -2  3 or -3  4 or -4  ≥5 or ≤-5 | 4879 (40.1)  4655 (38.3)  2624 (21.6)  3953 (32.5)  1480 (12.2)  605 (5.0)  321 (2.6)  920 (7.6) | 4614 (42.4)  3737 (34.4)  2521 (23.2)  3819 (35.1)  1442 (13.23)  580 (5.3)  135 (1.2)  282 (2.6) | 265 (20.6)  918 (71.4)  103 (8.0)  134 (10.4)  38 (3.0)  25 (1.9)  186 (14.5)  638 (49.6) |
| **KDIGO G2 (eGFR=60-89 ml/min/1.73m^2^):** | | | | |
| **Number of pregnancies (%)** | Total | 5980 | - | - |
|  | …with HES-APC linkage | 3535 | - | - |
|  | …with matching pregnancy in HES-APC | 3266 | 3034 | 232 |
|  | ...with recorded clinical GAB | 2354 | 2122 | 232 |
| **Clinical GAB, percentiles** | 0% (minimum)  1%  5%  10%  20%  25%  30%  40%  50% (median)  60%  70%  75%  80%  90%  95%  99%  100% (maximum) | 2.0  26.0  35.0  37.0  38.0  38.0  38.0  39.0  39.0  40.0  40.0  40.0  40.0  41.0  41.0  42.0  45.0 | 37.0  37.0  37.0  38.0  38.0  38.0  39.0  39.0  39.0  40.0  40.0  40.0  40.0  41.0  41.0  42.0  45.0 | 2.0  5.86  12.0  26.0  29.0  31.0  33.0  34.0  34.0  35.0  36.0  36.0  36.0  36.0  36.0  36.0  36.0 |
| **Clinical GAB – estimated GAB, percentiles** | 0% (min)  1%  5%  10%  20%  25%  30%  40%  50% (median)  60%  70%  75%  80%  90%  95%  99%  100% (max) | -38.0  -13.0  -4.0  -3.0  -2.0  -1.0  -1.0  0.0  0.0  0.0  0.0  0.0  1.0  1.0  2.0  6.0  17.0 | -14.0  -4.0  -3.0  -2.0  -1.0  -1.0  -1.0  0.0  0.0  0.0  0.0  0.0  1.0  1.0  2.0  5.0  17.0 | -38.0  -34.76  -26.45  -13.0  -8.0  -6.25  -6.0  -5.0  -4.0  -1.0  0.0  0.0  0.0  0.0  1.0  7.69  16.0 |
| **Clinical GAB – estimated GAB, number of pregnancies (%)** | 0  negative  positive  1 or -1  2 or -2  3 or -3  4 or -4  ≥5 or ≤-5 | 1002 (42.6)  872 (37.0)  480 (20.4)  732 (31.1)  277 (11.8)  132 (5.6)  61 (2.6)  150 (6.4) | 942 (44.4)  721 (34.0)  459 (21.6)  708 (33.4)  269 (12.7)  126 (5.9)  26 (1.2)  51 (2.4) | 60 (25.9)  151 (65.1)  21 (9.1)  24 (10.3)  8 (3.5)  6 (2.6)  35 (15.1)  99 (42.7) |
| **KDIGO G3/G4 (eGFR=15-59 ml/min/1.73m^2^):** | | | | |
| **Number of pregnancies (%)** | Total | 132 | - | - |
|  | …with HES-APC linkage | 79 | - | - |
|  | …with matching pregnancy in HES-APC | 71 | 55 | 16 |
|  | ...with recorded clinical GAB | 53 | 37 | 16 |
| **Clinical GAB, percentiles** | 0% (minimum)  1%  5%  10%  20%  25%  30%  40%  50% (median)  60%  70%  75%  80%  90%  95%  99%  100% (maximum) | 26.0  26.5  28.0  33.2  35.0  36.0  36.6  37.0  38.0  39.0  40.0  40.0  40.6  41.0  41.0  42.0  42.0 | 37.0  37.0  37.0  37.0  38.0  38.0  38.0  38.4  39.0  40.0  40.2  41.0  41.0  41.0  41.2  42.0  42.0 | 26.0  26.2  26.8  27.5  28.0  29.5  31.5  34.0  34.0  35.0  35.0  35.3  36.0  36.0  36.0  36.0  36.0 |
| **Clinical GAB – estimated GAB, percentiles** | 0% (min)  1%  5%  10%  20%  25%  30%  40%  50% (median)  60%  70%  75%  80%  90%  95%  99%  100% (max) | -14.0  -13.0  -10.8  -5.0  -3.0  -2.0  -2.0  -1.0  0.0  0.0  0.0  0.0  1.0  1.0  2.0  7.0  7.0 | -3.0  -3.0  -3.0  -2.4  -1.8  -1.0  -1.0  0.0  0.0  0.0  0.0  0.0  1.0  1.0  2.0  5.2  7.0 | -14.0  -13.7  -12.5  -12.0  -10.0  -7.8  -6.0  -5.0  -4.5  0.0  0.0  0.0  0.0  1.0  2.5  6.1  7.0 |
| **Clinical GAB – estimated GAB, number of pregnancies (%)** | 0  negative  positive  1 or -1  2 or -2  3 or -3  4 or -4  ≥5 or ≤-5 | 19 (35.9)  22 (41.5)  12 (22.6)  13 (24.5)  6 (11.3)  4 (7.6)  1 (1.9)  10 (18.9) | 15 (40.5)  13 (35.1)  9 (24.3)  11 (29.7)  6 (16.2)  4 (10.8)  0 (0.0)  1 (2.7) | 4 (25.0)  9 (56.3)  3 (18.8)  2 (12.5)  0 (0.0)  0 (0.0)  1 (6.3)  9 (56.3) |

Figure S1: Distribution of clinical GAB minus estimated GAB in pregnant females during validation of the pregnancy start date in CPRD GOLD. Negative numbers on the horizontal axis represent estimated GAB longer than clinical GAB; positive numbers represent estimated GAB shorter than clinical GAB.

|  | **Term and preterm delivery** | **Term delivery**^[[4]](#footnote-4)^ | **Preterm delivery**^[[5]](#footnote-5)^ |
| --- | --- | --- | --- |
| **Overall** | 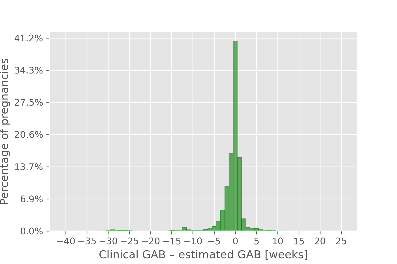 | 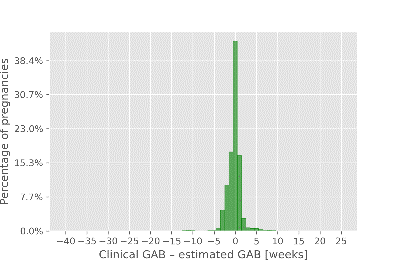 | 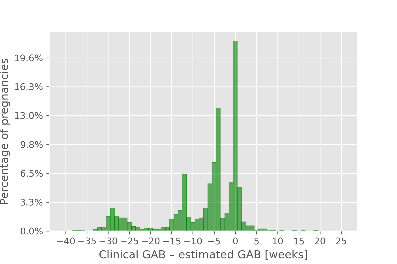 |
| **KDIGO G1** (eGFR ≥ 90 ml/min/1.73 m^2^) | 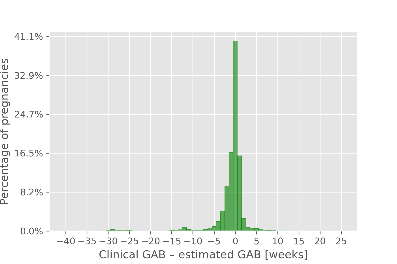 | 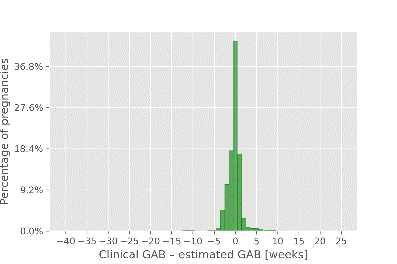 | 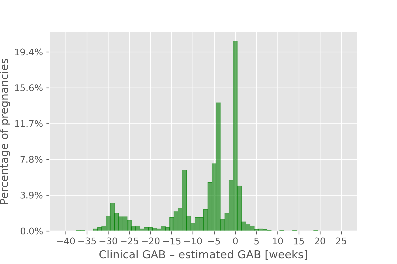 |
| **KDIGO G2** (eGFR = 60-89 ml/min/1.73 m^2^) | 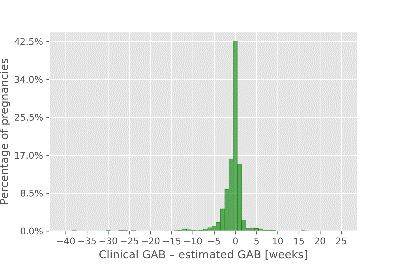 | 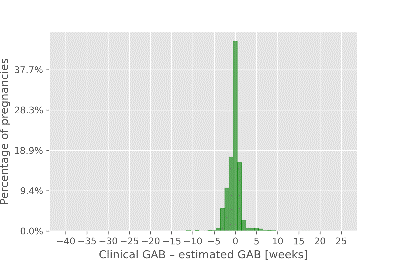 | 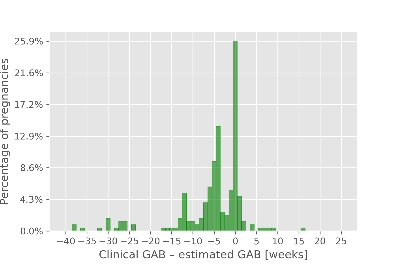 |
| **KDIGO G3/G4** (eGFR = 15-59 ml/min/1.73 m^2^) | 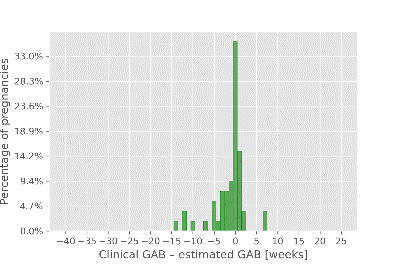 | 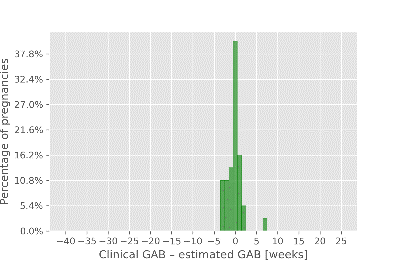 | 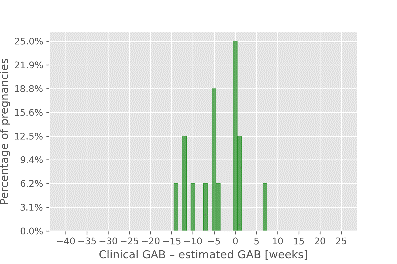 |

## Extraction of serum creatinine

### Overview

Serum creatinine (SCr) measurement with the unit µmol/L were considered and SCr levels between 1 and 1000 µmol/L. Identical SCr levels recorded on the same day were counted as a single SCr measurement.

### Read codes used to identify SCr in CPRD

**Table S2:** Read codes considered for the extraction of SCr levels

| Read code | Description |
| --- | --- |
| 44J3.00 | Serum creatinine |
| 44JF.00 | Plasma creatinine level |
| 44J3200 | Serum creatinine normal |
| 44J3z00 | Serum creatinine NOS |
| 44J3300 | Serum creatinine raised |
| 44JD.00 | Corrected serum creatinine level |
| 44JC.00 | Corrected plasma creatinine level |
| 44J3100 | Serum creatinine low |
| 44J3000 | Serum creatinine abnormal |

## Categorization of pregnancies into KDIGO G-categories

Due to under-recording of albuminuria, identification of CKD as suggested by the ‘Kidney Disease: Improving Global Outcomes’ (KDIGO) guideline is not possible in CPRD GOLD. Therefore, we identified reduced renal filtration prior to pregnancy (closest possible approximation of CKD in pregnancy in CPRD GOLD) by eGFR based on ≥1 recorded SCr level during baseline/trimester 1 using the 2009 CKD Epidemiology Collaboration (CKD-EPI) creatinine equation, assuming Caucasian ethnicity for missing data (37.3%).

We estimated the median baseline eGFR in all pregnancies separately based on all SCr levels measured during baseline/trimester 1. We also considered SCr levels measured during trimester 1, because some patients do not obtain blood work until their pregnancy comes to medical attention. We categorized pregnancies based on the KDIGO G-categories as follows: 1) normal or high eGFR [ml/min/1.73m^2^] ≥90 (G1), 2) mildly decreased eGFR=60-89 (G2), or 3) mildly to severely decreased eGFR=15-59 (G3/G4). We divided eGFR=60-89 (G2) into two groups post-hoc (G2-high vs. G2-low, only one category in KDIGO guidelines) as sample size was large enough to gain insights into those with a baseline eGFR between 60 and 74 vs. between 75 and 89 ml/min/1.73m^2^. To minimize non-differential misclassification, we excluded pregnancies that were categorized as G1, but had a Read code for any renal disease.

## Covariables

### Overview

Pre-specified baseline covariables included demographics, renal filtration, smoking status, alcohol abuse, and ethnicity. We further captured the absolute number and the median number of SCr measurements per pregnancy in baseline/trimester 1 and during the observation period. We captured pre-existing comorbidities and risk factors for CKD. Evaluated comorbidities included metabolic causes of CKD: Diabetes was defined as pre-existing if ≥1 diagnosis (Dx) code for diabetes and/or ≥1 prescription (Rx) for an antidiabetic drug (Table S3) and/or an HbA1c level ≥6.5% was recorded. Pre-existing hypertension was defined as ≥1 Dx code for hypertension and/or ≥1 Rx for an antihypertensive drug (Table S3). Comorbidities included systemic autoimmune disorders, type of kidney diseases (immune-mediated and non-immune-mediated), and other causes of CKD.

We further captured materno-fetal outcomes recorded in HES-APC (type of delivery, GAB, birth weight).

**Table S3:** Antidiabetic and antihypertensive drugs used as part of the definition of diabetes and hypertension

| Antidiabetic drugs | Alpha-glucosidase inhibitors (AGI) |
| --- | --- |
|  | Biguanides |
|  | Dipeptidyl peptidase-4 (DPP-4) inhibitor |
|  | Glinides |
|  | Glucagon-like peptide-1 (GLP-1) analogues |
|  | Insulin |
|  | Sodium-glucose co-transporter 2 (SGLT2) inhibitors |
|  | Sulfonylureas |
|  | Thiazolidinediones (TZD) |
|  | Combinations of the above mentioned antidiabetic drugs |
| Anti-hypertensive drugs | Alpha-adrenoreceptor antagonists |
|  | Angiotensin-converting enzyme (ACE) inhibitors with/without diuretics |
|  | Angiotensin II receptor blockers with/without diuretics |
|  | Beta-blockers with/without thiazides |
|  | Calcium channel blockers with/without diuretics |
|  | Renin inhibitors |

## Statistical analyses

Analyses were conducted using Python 3.0.^4,5^

# **Further results**

## Numeric information for Figure 1 (changes in SCr levels over time)

**Table S4:** Final (linked) study population: Median SCr levels [mg/dL*] with percentiles and number of pregnancies in each time period (i.e., between period start and period end) by presence and severity of reduced renal filtration (cat = 1: G3/G4 (eGFR=15-59 ml/min/1.73 m²), cat = 2: G2-low (eGFR=60-74 ml/min/1.73 m²), cat = 3: G2-high (eGFR=75-89 ml/min/1.73 m²), cat = 4: G1 (eGFR≥ 90 ml/min/1.73 m^2^). *Conversion factor for µmol/L: multiplication by 88.4

| **cat** | **start_**  **day** | **end_**  **day** | **start_**  **week** | **end_**  **week** | **n_**  **preg** | **n_**  **scr** | **scr_**  **median** | **p0** | **p25** | **p75** | **p100** |
| --- | --- | --- | --- | --- | --- | --- | --- | --- | --- | --- | --- |
| 1 | -420 | -350 | -60 | -50 | 3 | 3 | 1.29 | 1.29 | 1.29 | 1.54 | 1.80 |
| 1 | -350 | -280 | -50 | -40 | 12 | 12 | 1.40 | 1.17 | 1.24 | 1.61 | 1.95 |
| 1 | -280 | -210 | -40 | -30 | 11 | 13 | 1.44 | 1.20 | 1.26 | 2.04 | 2.59 |
| 1 | -210 | -140 | -30 | -20 | 11 | 15 | 1.48 | 1.07 | 1.28 | 1.73 | 2.22 |
| 1 | -140 | -70 | -20 | -10 | 19 | 22 | 1.41 | 1.15 | 1.25 | 1.71 | 3.35 |
| 1 | -70 | 0 | -10 | 0 | 15 | 17 | 1.48 | 1.22 | 1.33 | 1.83 | 3.39 |
| 1 | 0 | 70 | 0 | 10 | 17 | 18 | 1.33 | 1.14 | 1.22 | 1.54 | 2.22 |
| 1 | 70 | 140 | 10 | 20 | 12 | 17 | 1.29 | 1.20 | 1.23 | 1.32 | 2.04 |
| 1 | 140 | 210 | 20 | 30 | 13 | 18 | 1.23 | 0.81 | 0.97 | 1.78 | 2.29 |
| 1 | 210 | 280 | 30 | 40 | 16 | 33 | 1.71 | 0.74 | 1.32 | 2.36 | 3.40 |
| 1 | 280 | 350 | 40 | 50 | 20 | 22 | 1.49 | 0.72 | 1.19 | 1.69 | 2.59 |
| 1 | 350 | 420 | 50 | 60 | 18 | 20 | 1.48 | 0.85 | 1.18 | 1.78 | 2.53 |
| 1 | 420 | 490 | 60 | 70 | 8 | 10 | 1.43 | 1.09 | 1.31 | 1.60 | 3.29 |
| 1 | 490 | 560 | 70 | 80 | 10 | 12 | 1.53 | 0.79 | 1.06 | 2.02 | 3.01 |
| 1 | 560 | 630 | 80 | 90 | 12 | 15 | 1.73 | 1.11 | 1.31 | 2.73 | 3.17 |
| 1 | 630 | 700 | 90 | 100 | 0 | 0 |  |  |  |  |  |
| 2 | -378 | -364 | -54 | -52 | 7 | 7 | 1.02 | 0.94 | 1.00 | 1.05 | 1.10 |
| 2 | -364 | -350 | -52 | -50 | 15 | 15 | 1.06 | 0.95 | 1.03 | 1.10 | 1.18 |
| 2 | -350 | -336 | -50 | -48 | 12 | 12 | 1.02 | 0.98 | 0.99 | 1.06 | 1.19 |
| 2 | -336 | -322 | -48 | -46 | 19 | 19 | 1.06 | 0.96 | 1.02 | 1.10 | 1.55 |
| 2 | -322 | -308 | -46 | -44 | 18 | 19 | 1.09 | 0.95 | 1.02 | 1.14 | 1.38 |
| 2 | -308 | -294 | -44 | -42 | 16 | 16 | 1.07 | 1.00 | 1.03 | 1.14 | 1.23 |
| 2 | -294 | -280 | -42 | -40 | 10 | 10 | 1.04 | 0.98 | 1.01 | 1.09 | 1.20 |
| 2 | -280 | -266 | -40 | -38 | 18 | 18 | 1.04 | 0.93 | 1.00 | 1.11 | 1.43 |
| 2 | -266 | -252 | -38 | -36 | 17 | 18 | 1.04 | 0.86 | 1.02 | 1.11 | 1.27 |
| 2 | -252 | -238 | -36 | -34 | 21 | 22 | 1.04 | 0.89 | 0.97 | 1.06 | 1.13 |
| 2 | -238 | -224 | -34 | -32 | 21 | 21 | 1.07 | 0.98 | 1.03 | 1.11 | 1.19 |
| 2 | -224 | -210 | -32 | -30 | 21 | 21 | 1.05 | 0.89 | 1.02 | 1.12 | 1.19 |
| 2 | -210 | -196 | -30 | -28 | 19 | 19 | 1.05 | 1.00 | 1.02 | 1.09 | 1.20 |
| 2 | -196 | -182 | -28 | -26 | 10 | 10 | 1.06 | 1.00 | 1.02 | 1.11 | 1.21 |
| 2 | -182 | -168 | -26 | -24 | 14 | 14 | 1.06 | 0.97 | 1.01 | 1.10 | 1.18 |
| 2 | -168 | -154 | -24 | -22 | 24 | 24 | 1.06 | 0.92 | 1.03 | 1.13 | 1.26 |
| 2 | -154 | -140 | -22 | -20 | 24 | 24 | 1.03 | 0.93 | 0.99 | 1.06 | 1.13 |
| 2 | -140 | -126 | -20 | -18 | 19 | 19 | 1.04 | 0.85 | 1.01 | 1.06 | 1.24 |
| 2 | -126 | -112 | -18 | -16 | 18 | 18 | 1.06 | 0.98 | 1.02 | 1.13 | 1.23 |
| 2 | -112 | -98 | -16 | -14 | 23 | 25 | 1.03 | 0.86 | 0.98 | 1.06 | 1.47 |
| 2 | -98 | -84 | -14 | -12 | 20 | 20 | 1.05 | 0.86 | 1.02 | 1.09 | 1.12 |
| 2 | -84 | -70 | -12 | -10 | 22 | 23 | 1.02 | 0.92 | 1.00 | 1.05 | 1.28 |
| 2 | -70 | -56 | -10 | -8 | 22 | 22 | 1.05 | 0.94 | 1.00 | 1.09 | 1.20 |
| 2 | -56 | -42 | -8 | -6 | 22 | 22 | 1.05 | 0.90 | 1.00 | 1.10 | 1.19 |
| 2 | -42 | -28 | -6 | -4 | 26 | 28 | 1.04 | 0.95 | 1.01 | 1.09 | 1.15 |
| 2 | -28 | -14 | -4 | -2 | 18 | 18 | 1.07 | 0.98 | 1.00 | 1.13 | 1.20 |
| 2 | -14 | 0 | -2 | 0 | 16 | 17 | 1.07 | 1.00 | 1.04 | 1.10 | 1.15 |
| 2 | 0 | 14 | 0 | 2 | 25 | 25 | 1.05 | 0.93 | 1.03 | 1.07 | 1.12 |
| 2 | 14 | 28 | 2 | 4 | 20 | 20 | 1.07 | 0.86 | 1.02 | 1.14 | 1.23 |
| 2 | 28 | 42 | 4 | 6 | 21 | 22 | 1.05 | 0.81 | 1.00 | 1.09 | 1.41 |
| 2 | 42 | 56 | 6 | 8 | 4 | 4 | 1.01 | 0.97 | 0.98 | 1.04 | 1.09 |
| 2 | 56 | 70 | 8 | 10 | 5 | 5 | 0.96 | 0.93 | 0.95 | 0.98 | 1.00 |
| 2 | 70 | 84 | 10 | 12 | 5 | 5 | 0.94 | 0.81 | 0.88 | 1.06 | 1.13 |
| 2 | 84 | 98 | 12 | 14 | 6 | 6 | 0.84 | 0.48 | 0.58 | 0.94 | 1.04 |
| 2 | 98 | 112 | 14 | 16 | 10 | 10 | 0.81 | 0.60 | 0.66 | 0.89 | 1.05 |
| 2 | 112 | 126 | 16 | 18 | 8 | 8 | 0.79 | 0.63 | 0.74 | 0.86 | 1.09 |
| 2 | 126 | 140 | 18 | 20 | 6 | 6 | 0.77 | 0.53 | 0.69 | 0.87 | 0.97 |
| 2 | 140 | 154 | 20 | 22 | 7 | 7 | 0.85 | 0.78 | 0.83 | 0.88 | 1.05 |
| 2 | 154 | 168 | 22 | 24 | 7 | 7 | 0.67 | 0.53 | 0.57 | 0.77 | 1.04 |
| 2 | 168 | 182 | 24 | 26 | 15 | 16 | 0.79 | 0.66 | 0.70 | 0.83 | 0.94 |
| 2 | 182 | 196 | 26 | 28 | 20 | 20 | 0.77 | 0.62 | 0.69 | 0.83 | 0.90 |
| 2 | 196 | 210 | 28 | 30 | 11 | 11 | 0.80 | 0.51 | 0.71 | 0.85 | 0.88 |
| 2 | 210 | 224 | 30 | 32 | 8 | 8 | 0.81 | 0.67 | 0.74 | 0.91 | 1.12 |
| 2 | 224 | 238 | 32 | 34 | 17 | 20 | 0.74 | 0.51 | 0.64 | 0.93 | 1.44 |
| 2 | 238 | 252 | 34 | 36 | 22 | 28 | 0.81 | 0.45 | 0.71 | 0.97 | 1.62 |
| 2 | 252 | 266 | 36 | 38 | 20 | 20 | 0.88 | 0.49 | 0.83 | 1.00 | 1.46 |
| 2 | 266 | 280 | 38 | 40 | 23 | 26 | 0.85 | 0.59 | 0.73 | 1.02 | 1.20 |
| 2 | 280 | 294 | 40 | 42 | 14 | 15 | 0.94 | 0.53 | 0.76 | 1.05 | 1.12 |
| 2 | 294 | 308 | 42 | 44 | 19 | 19 | 0.98 | 0.70 | 0.94 | 1.08 | 1.35 |
| 2 | 308 | 322 | 44 | 46 | 17 | 17 | 1.02 | 0.74 | 0.95 | 1.11 | 1.41 |
| 2 | 322 | 336 | 46 | 48 | 16 | 17 | 1.05 | 0.81 | 1.03 | 1.13 | 1.31 |
| 2 | 336 | 350 | 48 | 50 | 19 | 21 | 1.07 | 0.69 | 0.93 | 1.18 | 1.29 |
| 2 | 350 | 364 | 50 | 52 | 13 | 13 | 1.00 | 0.72 | 0.94 | 1.06 | 1.47 |
| 2 | 364 | 378 | 52 | 54 | 15 | 16 | 0.93 | 0.79 | 0.88 | 1.02 | 1.45 |
| 2 | 378 | 392 | 54 | 56 | 11 | 12 | 1.01 | 0.89 | 0.99 | 1.09 | 1.26 |
| 2 | 392 | 406 | 56 | 58 | 12 | 14 | 1.00 | 0.81 | 0.90 | 1.04 | 1.14 |
| 2 | 406 | 420 | 58 | 60 | 22 | 23 | 1.01 | 0.64 | 0.87 | 1.09 | 1.44 |
| 2 | 420 | 434 | 60 | 62 | 17 | 17 | 0.95 | 0.70 | 0.92 | 1.04 | 1.14 |
| 2 | 434 | 448 | 62 | 64 | 10 | 10 | 1.06 | 0.78 | 0.90 | 1.15 | 1.19 |
| 2 | 448 | 462 | 64 | 66 | 19 | 19 | 0.96 | 0.70 | 0.88 | 1.06 | 1.40 |
| 2 | 462 | 476 | 66 | 68 | 13 | 13 | 1.01 | 0.71 | 0.92 | 1.09 | 1.19 |
| 2 | 476 | 490 | 68 | 70 | 7 | 7 | 0.89 | 0.60 | 0.66 | 1.01 | 1.02 |
| 2 | 490 | 504 | 70 | 72 | 22 | 22 | 0.98 | 0.78 | 0.94 | 1.05 | 1.13 |
| 2 | 504 | 518 | 72 | 74 | 11 | 11 | 0.98 | 0.90 | 0.96 | 1.04 | 1.11 |
| 2 | 518 | 532 | 74 | 76 | 10 | 10 | 0.92 | 0.67 | 0.85 | 0.97 | 1.10 |
| 2 | 532 | 546 | 76 | 78 | 12 | 12 | 0.94 | 0.69 | 0.82 | 1.05 | 1.14 |
| 2 | 546 | 560 | 78 | 80 | 13 | 14 | 0.93 | 0.61 | 0.81 | 1.00 | 1.24 |
| 2 | 560 | 574 | 80 | 82 | 14 | 14 | 0.89 | 0.60 | 0.76 | 0.97 | 1.05 |
| 2 | 574 | 588 | 82 | 84 | 12 | 12 | 0.96 | 0.83 | 0.87 | 1.07 | 1.19 |
| 2 | 588 | 602 | 84 | 86 | 10 | 10 | 0.93 | 0.72 | 0.83 | 1.09 | 1.13 |
| 2 | 602 | 616 | 86 | 88 | 9 | 10 | 1.01 | 0.74 | 0.84 | 1.03 | 1.12 |
| 2 | 616 | 630 | 88 | 90 | 12 | 12 | 0.95 | 0.79 | 0.85 | 1.05 | 1.31 |
| 2 | 630 | 644 | 90 | 92 | 5 | 5 | 0.94 | 0.72 | 0.90 | 0.94 | 1.09 |
| 2 | 644 | 658 | 92 | 94 | 3 | 3 | 0.98 | 0.77 | 0.88 | 1.22 | 1.46 |
| 3 | -378 | -364 | -54 | -52 | 25 | 25 | 0.93 | 0.76 | 0.89 | 0.95 | 1.03 |
| 3 | -364 | -350 | -52 | -50 | 78 | 87 | 0.92 | 0.74 | 0.88 | 0.98 | 1.41 |
| 3 | -350 | -336 | -50 | -48 | 94 | 102 | 0.90 | 0.69 | 0.87 | 0.96 | 1.14 |
| 3 | -336 | -322 | -48 | -46 | 78 | 79 | 0.93 | 0.77 | 0.89 | 0.97 | 1.46 |
| 3 | -322 | -308 | -46 | -44 | 104 | 109 | 0.90 | 0.78 | 0.87 | 0.95 | 1.17 |
| 3 | -308 | -294 | -44 | -42 | 96 | 96 | 0.93 | 0.72 | 0.89 | 0.96 | 1.07 |
| 3 | -294 | -280 | -42 | -40 | 90 | 91 | 0.93 | 0.76 | 0.89 | 0.96 | 1.14 |
| 3 | -280 | -266 | -40 | -38 | 98 | 100 | 0.92 | 0.76 | 0.88 | 0.96 | 1.16 |
| 3 | -266 | -252 | -38 | -36 | 82 | 84 | 0.93 | 0.77 | 0.89 | 0.96 | 1.09 |
| 3 | -252 | -238 | -36 | -34 | 95 | 95 | 0.92 | 0.77 | 0.88 | 0.96 | 1.10 |
| 3 | -238 | -224 | -34 | -32 | 93 | 93 | 0.94 | 0.74 | 0.88 | 0.96 | 1.17 |
| 3 | -224 | -210 | -32 | -30 | 96 | 96 | 0.92 | 0.80 | 0.89 | 0.96 | 1.24 |
| 3 | -210 | -196 | -30 | -28 | 96 | 97 | 0.93 | 0.78 | 0.88 | 0.97 | 1.17 |
| 3 | -196 | -182 | -28 | -26 | 91 | 94 | 0.93 | 0.78 | 0.89 | 0.97 | 1.49 |
| 3 | -182 | -168 | -26 | -24 | 81 | 83 | 0.92 | 0.75 | 0.88 | 0.96 | 1.11 |
| 3 | -168 | -154 | -24 | -22 | 86 | 86 | 0.92 | 0.77 | 0.89 | 0.97 | 1.12 |
| 3 | -154 | -140 | -22 | -20 | 98 | 102 | 0.92 | 0.76 | 0.88 | 0.97 | 1.22 |
| 3 | -140 | -126 | -20 | -18 | 90 | 90 | 0.93 | 0.84 | 0.89 | 0.98 | 1.10 |
| 3 | -126 | -112 | -18 | -16 | 87 | 89 | 0.93 | 0.78 | 0.89 | 0.96 | 1.13 |
| 3 | -112 | -98 | -16 | -14 | 97 | 98 | 0.93 | 0.75 | 0.88 | 0.97 | 1.07 |
| 3 | -98 | -84 | -14 | -12 | 96 | 98 | 0.93 | 0.67 | 0.87 | 0.95 | 1.23 |
| 3 | -84 | -70 | -12 | -10 | 86 | 90 | 0.92 | 0.75 | 0.88 | 0.96 | 1.17 |
| 3 | -70 | -56 | -10 | -8 | 96 | 98 | 0.93 | 0.79 | 0.89 | 0.97 | 1.22 |
| 3 | -56 | -42 | -8 | -6 | 107 | 109 | 0.92 | 0.68 | 0.88 | 0.96 | 1.47 |
| 3 | -42 | -28 | -6 | -4 | 95 | 96 | 0.89 | 0.77 | 0.87 | 0.95 | 1.06 |
| 3 | -28 | -14 | -4 | -2 | 103 | 103 | 0.92 | 0.70 | 0.87 | 0.95 | 1.06 |
| 3 | -14 | 0 | -2 | 0 | 95 | 100 | 0.92 | 0.78 | 0.88 | 0.95 | 1.36 |
| 3 | 0 | 14 | 0 | 2 | 98 | 99 | 0.90 | 0.77 | 0.88 | 0.96 | 1.12 |
| 3 | 14 | 28 | 2 | 4 | 91 | 91 | 0.92 | 0.77 | 0.87 | 0.95 | 1.12 |
| 3 | 28 | 42 | 4 | 6 | 67 | 67 | 0.90 | 0.79 | 0.87 | 0.94 | 1.00 |
| 3 | 42 | 56 | 6 | 8 | 55 | 58 | 0.89 | 0.70 | 0.85 | 0.94 | 1.12 |
| 3 | 56 | 70 | 8 | 10 | 46 | 48 | 0.84 | 0.62 | 0.81 | 0.89 | 0.97 |
| 3 | 70 | 84 | 10 | 12 | 24 | 26 | 0.85 | 0.52 | 0.81 | 0.89 | 1.56 |
| 3 | 84 | 98 | 12 | 14 | 20 | 20 | 0.75 | 0.46 | 0.68 | 0.83 | 1.02 |
| 3 | 98 | 112 | 14 | 16 | 49 | 50 | 0.67 | 0.53 | 0.60 | 0.75 | 0.95 |
| 3 | 112 | 126 | 16 | 18 | 44 | 44 | 0.71 | 0.52 | 0.66 | 0.77 | 1.00 |
| 3 | 126 | 140 | 18 | 20 | 35 | 36 | 0.70 | 0.48 | 0.60 | 0.79 | 1.01 |
| 3 | 140 | 154 | 20 | 22 | 33 | 33 | 0.68 | 0.50 | 0.62 | 0.72 | 0.89 |
| 3 | 154 | 168 | 22 | 24 | 55 | 55 | 0.68 | 0.48 | 0.61 | 0.76 | 1.01 |
| 3 | 168 | 182 | 24 | 26 | 42 | 42 | 0.64 | 0.49 | 0.58 | 0.72 | 0.90 |
| 3 | 182 | 196 | 26 | 28 | 58 | 58 | 0.68 | 0.45 | 0.59 | 0.77 | 0.95 |
| 3 | 196 | 210 | 28 | 30 | 60 | 64 | 0.66 | 0.44 | 0.59 | 0.72 | 1.00 |
| 3 | 210 | 224 | 30 | 32 | 56 | 59 | 0.67 | 0.43 | 0.62 | 0.75 | 1.14 |
| 3 | 224 | 238 | 32 | 34 | 74 | 76 | 0.70 | 0.43 | 0.60 | 0.76 | 1.01 |
| 3 | 238 | 252 | 34 | 36 | 64 | 69 | 0.70 | 0.33 | 0.63 | 0.78 | 1.06 |
| 3 | 252 | 266 | 36 | 38 | 81 | 91 | 0.77 | 0.48 | 0.70 | 0.87 | 1.13 |
| 3 | 266 | 280 | 38 | 40 | 73 | 79 | 0.76 | 0.51 | 0.68 | 0.87 | 1.18 |
| 3 | 280 | 294 | 40 | 42 | 55 | 59 | 0.84 | 0.42 | 0.74 | 0.90 | 1.10 |
| 3 | 294 | 308 | 42 | 44 | 51 | 51 | 0.94 | 0.68 | 0.83 | 0.99 | 1.40 |
| 3 | 308 | 322 | 44 | 46 | 73 | 74 | 0.93 | 0.60 | 0.85 | 1.00 | 1.21 |
| 3 | 322 | 336 | 46 | 48 | 70 | 71 | 0.92 | 0.60 | 0.83 | 0.96 | 1.33 |
| 3 | 336 | 350 | 48 | 50 | 59 | 61 | 0.89 | 0.62 | 0.84 | 0.97 | 1.14 |
| 3 | 350 | 364 | 50 | 52 | 54 | 55 | 0.89 | 0.53 | 0.80 | 1.00 | 1.18 |
| 3 | 364 | 378 | 52 | 54 | 42 | 43 | 0.87 | 0.54 | 0.80 | 0.96 | 1.24 |
| 3 | 378 | 392 | 54 | 56 | 71 | 73 | 0.89 | 0.63 | 0.80 | 0.94 | 1.06 |
| 3 | 392 | 406 | 56 | 58 | 71 | 71 | 0.88 | 0.54 | 0.76 | 0.95 | 1.10 |
| 3 | 406 | 420 | 58 | 60 | 55 | 55 | 0.85 | 0.66 | 0.77 | 0.90 | 1.10 |
| 3 | 420 | 434 | 60 | 62 | 60 | 60 | 0.85 | 0.52 | 0.79 | 0.92 | 1.10 |
| 3 | 434 | 448 | 62 | 64 | 54 | 54 | 0.88 | 0.59 | 0.79 | 0.93 | 1.28 |
| 3 | 448 | 462 | 64 | 66 | 68 | 69 | 0.90 | 0.66 | 0.84 | 0.96 | 1.19 |
| 3 | 462 | 476 | 66 | 68 | 58 | 60 | 0.87 | 0.54 | 0.80 | 0.96 | 1.14 |
| 3 | 476 | 490 | 68 | 70 | 52 | 52 | 0.86 | 0.63 | 0.79 | 0.95 | 1.36 |
| 3 | 490 | 504 | 70 | 72 | 47 | 48 | 0.83 | 0.63 | 0.76 | 0.92 | 1.09 |
| 3 | 504 | 518 | 72 | 74 | 44 | 45 | 0.88 | 0.50 | 0.77 | 0.96 | 1.04 |
| 3 | 518 | 532 | 74 | 76 | 64 | 65 | 0.85 | 0.59 | 0.79 | 0.89 | 1.13 |
| 3 | 532 | 546 | 76 | 78 | 50 | 51 | 0.87 | 0.54 | 0.79 | 0.93 | 1.14 |
| 3 | 546 | 560 | 78 | 80 | 52 | 55 | 0.85 | 0.53 | 0.78 | 0.94 | 1.11 |
| 3 | 560 | 574 | 80 | 82 | 67 | 68 | 0.86 | 0.53 | 0.79 | 0.92 | 1.11 |
| 3 | 574 | 588 | 82 | 84 | 39 | 39 | 0.86 | 0.61 | 0.81 | 0.89 | 1.20 |
| 3 | 588 | 602 | 84 | 86 | 59 | 62 | 0.85 | 0.61 | 0.75 | 0.92 | 1.11 |
| 3 | 602 | 616 | 86 | 88 | 60 | 62 | 0.87 | 0.66 | 0.79 | 0.92 | 1.10 |
| 3 | 616 | 630 | 88 | 90 | 38 | 39 | 0.84 | 0.61 | 0.74 | 0.88 | 1.10 |
| 3 | 630 | 644 | 90 | 92 | 41 | 41 | 0.84 | 0.60 | 0.72 | 0.89 | 1.07 |
| 3 | 644 | 658 | 92 | 94 | 12 | 13 | 0.77 | 0.68 | 0.74 | 0.88 | 1.13 |
| 4 | -378 | -364 | -54 | -52 | 180 | 182 | 0.71 | 0.45 | 0.64 | 0.79 | 1.09 |
| 4 | -364 | -350 | -52 | -50 | 491 | 497 | 0.74 | 0.31 | 0.67 | 0.80 | 1.02 |
| 4 | -350 | -336 | -50 | -48 | 513 | 519 | 0.74 | 0.34 | 0.66 | 0.80 | 1.09 |
| 4 | -336 | -322 | -48 | -46 | 508 | 514 | 0.74 | 0.45 | 0.67 | 0.80 | 1.03 |
| 4 | -322 | -308 | -46 | -44 | 520 | 525 | 0.74 | 0.32 | 0.66 | 0.80 | 1.02 |
| 4 | -308 | -294 | -44 | -42 | 529 | 538 | 0.74 | 0.41 | 0.67 | 0.80 | 1.09 |
| 4 | -294 | -280 | -42 | -40 | 510 | 513 | 0.71 | 0.04 | 0.66 | 0.79 | 1.15 |
| 4 | -280 | -266 | -40 | -38 | 517 | 525 | 0.72 | 0.35 | 0.64 | 0.79 | 1.00 |
| 4 | -266 | -252 | -38 | -36 | 522 | 530 | 0.74 | 0.41 | 0.67 | 0.80 | 1.10 |
| 4 | -252 | -238 | -36 | -34 | 513 | 515 | 0.72 | 0.09 | 0.67 | 0.80 | 1.02 |
| 4 | -238 | -224 | -34 | -32 | 564 | 579 | 0.72 | 0.32 | 0.66 | 0.79 | 1.14 |
| 4 | -224 | -210 | -32 | -30 | 517 | 526 | 0.72 | 0.45 | 0.67 | 0.80 | 1.13 |
| 4 | -210 | -196 | -30 | -28 | 542 | 554 | 0.74 | 0.05 | 0.66 | 0.80 | 1.03 |
| 4 | -196 | -182 | -28 | -26 | 487 | 491 | 0.72 | 0.09 | 0.66 | 0.79 | 1.13 |
| 4 | -182 | -168 | -26 | -24 | 552 | 560 | 0.72 | 0.18 | 0.64 | 0.79 | 1.03 |
| 4 | -168 | -154 | -24 | -22 | 533 | 541 | 0.72 | 0.38 | 0.64 | 0.78 | 1.00 |
| 4 | -154 | -140 | -22 | -20 | 557 | 568 | 0.72 | 0.23 | 0.66 | 0.79 | 1.06 |
| 4 | -140 | -126 | -20 | -18 | 547 | 558 | 0.71 | 0.36 | 0.66 | 0.78 | 1.02 |
| 4 | -126 | -112 | -18 | -16 | 547 | 558 | 0.72 | 0.42 | 0.67 | 0.79 | 1.06 |
| 4 | -112 | -98 | -16 | -14 | 593 | 607 | 0.71 | 0.41 | 0.64 | 0.79 | 1.14 |
| 4 | -98 | -84 | -14 | -12 | 537 | 546 | 0.73 | 0.20 | 0.66 | 0.79 | 1.01 |
| 4 | -84 | -70 | -12 | -10 | 569 | 581 | 0.71 | 0.33 | 0.64 | 0.78 | 1.22 |
| 4 | -70 | -56 | -10 | -8 | 620 | 629 | 0.72 | 0.10 | 0.66 | 0.79 | 1.05 |
| 4 | -56 | -42 | -8 | -6 | 583 | 592 | 0.72 | 0.42 | 0.66 | 0.78 | 0.95 |
| 4 | -42 | -28 | -6 | -4 | 576 | 587 | 0.71 | 0.09 | 0.64 | 0.78 | 0.95 |
| 4 | -28 | -14 | -4 | -2 | 618 | 627 | 0.72 | 0.09 | 0.66 | 0.78 | 1.00 |
| 4 | -14 | 0 | -2 | 0 | 625 | 632 | 0.72 | 0.44 | 0.66 | 0.79 | 0.97 |
| 4 | 0 | 14 | 0 | 2 | 576 | 580 | 0.71 | 0.26 | 0.66 | 0.78 | 1.12 |
| 4 | 14 | 28 | 2 | 4 | 597 | 604 | 0.71 | 0.36 | 0.63 | 0.77 | 1.11 |
| 4 | 28 | 42 | 4 | 6 | 703 | 716 | 0.68 | 0.31 | 0.61 | 0.75 | 1.20 |
| 4 | 42 | 56 | 6 | 8 | 781 | 805 | 0.64 | 0.09 | 0.58 | 0.71 | 1.33 |
| 4 | 56 | 70 | 8 | 10 | 696 | 715 | 0.61 | 0.38 | 0.54 | 0.68 | 0.95 |
| 4 | 70 | 84 | 10 | 12 | 530 | 547 | 0.59 | 0.34 | 0.53 | 0.68 | 0.94 |
| 4 | 84 | 98 | 12 | 14 | 400 | 416 | 0.58 | 0.33 | 0.52 | 0.63 | 0.90 |
| 4 | 98 | 112 | 14 | 16 | 292 | 298 | 0.55 | 0.28 | 0.50 | 0.62 | 0.97 |
| 4 | 112 | 126 | 16 | 18 | 297 | 304 | 0.55 | 0.24 | 0.49 | 0.61 | 1.01 |
| 4 | 126 | 140 | 18 | 20 | 324 | 329 | 0.55 | 0.31 | 0.49 | 0.62 | 1.74 |
| 4 | 140 | 154 | 20 | 22 | 276 | 280 | 0.54 | 0.29 | 0.49 | 0.60 | 0.98 |
| 4 | 154 | 168 | 22 | 24 | 336 | 341 | 0.55 | 0.31 | 0.51 | 0.61 | 0.87 |
| 4 | 168 | 182 | 24 | 26 | 334 | 347 | 0.54 | 0.32 | 0.49 | 0.61 | 1.31 |
| 4 | 182 | 196 | 26 | 28 | 388 | 397 | 0.54 | 0.31 | 0.49 | 0.61 | 1.20 |
| 4 | 196 | 210 | 28 | 30 | 372 | 387 | 0.55 | 0.28 | 0.49 | 0.63 | 1.18 |
| 4 | 210 | 224 | 30 | 32 | 347 | 359 | 0.54 | 0.26 | 0.49 | 0.61 | 1.32 |
| 4 | 224 | 238 | 32 | 34 | 426 | 442 | 0.57 | 0.31 | 0.50 | 0.63 | 0.98 |
| 4 | 238 | 252 | 34 | 36 | 445 | 468 | 0.58 | 0.28 | 0.51 | 0.68 | 1.02 |
| 4 | 252 | 266 | 36 | 38 | 455 | 482 | 0.61 | 0.33 | 0.53 | 0.69 | 1.01 |
| 4 | 266 | 280 | 38 | 40 | 400 | 425 | 0.66 | 0.36 | 0.57 | 0.74 | 1.41 |
| 4 | 280 | 294 | 40 | 42 | 366 | 388 | 0.71 | 0.33 | 0.61 | 0.79 | 1.46 |
| 4 | 294 | 308 | 42 | 44 | 362 | 369 | 0.72 | 0.35 | 0.64 | 0.81 | 1.20 |
| 4 | 308 | 322 | 44 | 46 | 385 | 389 | 0.75 | 0.25 | 0.68 | 0.81 | 1.24 |
| 4 | 322 | 336 | 46 | 48 | 448 | 452 | 0.74 | 0.50 | 0.67 | 0.81 | 1.46 |
| 4 | 336 | 350 | 48 | 50 | 409 | 411 | 0.72 | 0.45 | 0.65 | 0.80 | 1.18 |
| 4 | 350 | 364 | 50 | 52 | 379 | 384 | 0.74 | 0.36 | 0.64 | 0.80 | 1.89 |
| 4 | 364 | 378 | 52 | 54 | 404 | 409 | 0.74 | 0.43 | 0.66 | 0.80 | 1.15 |
| 4 | 378 | 392 | 54 | 56 | 368 | 374 | 0.72 | 0.40 | 0.66 | 0.79 | 1.15 |
| 4 | 392 | 406 | 56 | 58 | 393 | 394 | 0.72 | 0.38 | 0.64 | 0.80 | 1.23 |
| 4 | 406 | 420 | 58 | 60 | 390 | 399 | 0.74 | 0.45 | 0.64 | 0.79 | 1.18 |
| 4 | 420 | 434 | 60 | 62 | 385 | 391 | 0.74 | 0.33 | 0.64 | 0.81 | 1.05 |
| 4 | 434 | 448 | 62 | 64 | 366 | 368 | 0.72 | 0.41 | 0.66 | 0.80 | 1.17 |
| 4 | 448 | 462 | 64 | 66 | 377 | 384 | 0.72 | 0.41 | 0.65 | 0.79 | 1.06 |
| 4 | 462 | 476 | 66 | 68 | 380 | 380 | 0.70 | 0.41 | 0.63 | 0.79 | 1.07 |
| 4 | 476 | 490 | 68 | 70 | 360 | 364 | 0.72 | 0.35 | 0.64 | 0.79 | 1.09 |
| 4 | 490 | 504 | 70 | 72 | 351 | 353 | 0.71 | 0.45 | 0.64 | 0.79 | 0.98 |
| 4 | 504 | 518 | 72 | 74 | 343 | 347 | 0.71 | 0.36 | 0.63 | 0.79 | 1.17 |
| 4 | 518 | 532 | 74 | 76 | 353 | 355 | 0.71 | 0.37 | 0.64 | 0.78 | 1.15 |
| 4 | 532 | 546 | 76 | 78 | 331 | 332 | 0.71 | 0.36 | 0.62 | 0.77 | 1.14 |
| 4 | 546 | 560 | 78 | 80 | 320 | 324 | 0.72 | 0.06 | 0.66 | 0.80 | 1.33 |
| 4 | 560 | 574 | 80 | 82 | 342 | 345 | 0.71 | 0.42 | 0.63 | 0.79 | 1.05 |
| 4 | 574 | 588 | 82 | 84 | 295 | 298 | 0.70 | 0.27 | 0.63 | 0.79 | 1.29 |
| 4 | 588 | 602 | 84 | 86 | 285 | 293 | 0.72 | 0.44 | 0.64 | 0.79 | 1.07 |
| 4 | 602 | 616 | 86 | 88 | 318 | 320 | 0.71 | 0.42 | 0.63 | 0.80 | 1.10 |
| 4 | 616 | 630 | 88 | 90 | 286 | 290 | 0.71 | 0.45 | 0.63 | 0.79 | 1.06 |
| 4 | 630 | 644 | 90 | 92 | 182 | 183 | 0.72 | 0.52 | 0.66 | 0.80 | 1.04 |
| 4 | 644 | 658 | 92 | 94 | 56 | 56 | 0.69 | 0.51 | 0.62 | 0.76 | 0.95 |

Abbreviations: cat = category, start_day = day of time period start, end_day = day of time period end, start_week = week of time period start, end_week = week of time period end, n_preg = number of pregnancy in corresponding time period, n_scr = number of SCr measurements in corresponding time period, scr_median = median SCr levels in corresponding time period, p0 = minimum (SCr level), p25 = 25^th^ percentile (SCr level), p75 = 75^th^ percentile (SCr level), p100 = maximum (SCr level)

## Baseline characteristics of CPRD GOLD population

**Table S5:** CPRD GOLD population: Baseline characteristics overall and by presence and severity of reduced baseline eGFR.

| Baseline characteristics | | **Overall** | **G1:**  **Normal or high eGFR ≥ 90 ml/min/1.73 m^2^** | **G2-high:**  **Mildly decreased eGFR=75-89 ml/min/1.73 m²** | **G2-low:**  **Mildly decreased eGFR=60-74 ml/min/1.73 m²** | **G3/G4:**  **Mildly to severely decreased eGFR=15-59 ml/min/1.73 m²** |
| --- | --- | --- | --- | --- | --- | --- |
| Number of pregnancies |  | 35’594 (100.0) | 30’324 (85.2) | 4’295 (12.1) | 858 (2.4) | 117 (0.3) |
| Number of pregnant females |  | 33’811 | 28’886 | 4’223 | 850 | 113 |
| eGFR [ml/min/1.73m^2^] in baseline^a^ / trimester 1^b^, median [IQR] |  | 112.03  [97.14,121.70] | 115.05 [104.11,123.14] | 83.96 [79.49,87.97] | 70.13  [65.88,73.42] | 52.99  [43.24,57.14] |
| SCr [mg/dL^c^] in baseline^a^/trimester 1^b^, median [IQR] |  | 0.72  [0.64,0.81] | 0.71  [0.63,0.78] | 0.92 [0.87,0.96] | 1.05 [1.01,1.11] | 1.36  [1.24,1.62] |
| Age at delivery [years], median [IQR] |  | 30.0 [26.0,35.0] | 30.0 [26.0,34.0] | 33.0 [29.0,36.0] | 34.0 [31.0,38.0] | 34.0 [30.0,38.0] |
| Calendar year of delivery | 2000-2004 | 2’161 (6.1) | 1’468 (4.8) | 518 (12.1) | 151 (17.6) | 24 (20.5) |
|  | 2005-2009 | 8’197 (23.0) | 6’075 (20.0) | 1’714 (39.9) | 363 (42.3) | 45 (38.5) |
|  | 2010-2014 | 14’662 (41.2) | 13’073 (43.1) | 1’333 (31.0) | 222 (25.9) | 34 (29.1) |
|  | 2015-2019 | 10’574 (29.7) | 9’708 (32.0) | 730 (17.0) | 122 (14.2) | 14 (12.0) |
| Time enrolled in CPRD GOLD before pregnancy start date [years], median [IQR] |  | 7.0 [2.6,14.6] | 7.0 [2.6,14.8] | 6.8 [2.6,13.6] | 6.2 [2.4,12.8] | 6.4 [1.9,13.1] |
| Ethnicity | Black | 3’002 (8.4) | 2’848 (9.4) | 127 (3.0) | 24 (2.8) | NR |
|  | White or other (except black) | 17’862 (50.2) | 15’193 (50.1) | 2’205 (51.3) | 409 (47.7) | 55 (47.0) |
|  | Unknown | 14’730 (41.4) | 12’283 (40.5) | 1’963 (45.7) | 425 (49.5) | 59 (50.4) |
| Current smoker^d^ |  | 5’167 (14.5) | 4’424 (14.6) | 607 (14.1) | 111 (12.9) | 25 (21.4) |
| Alcohol abuse^e^ |  | 1’167 (3.3) | 965 (3.2) | 163 (3.8) | 36 (4.2) | NR |
| Number of SCr measurements | Observation period^f^ | 100’827 | 85’985 | 11’732 | 2’583 | 527 |
|  | Per pregnancy in baseline^a^ / trimester 1^b^, median [IQR] | 1 [1, 2] | 1 [1, 2] | 1 [1, 2] | 1 [1, 2] | 2 [1, 2] |
|  | Per pregnancy in observation period^f^, median [IQR] | 2 [2, 3] | 2 [2, 3] | 2 [2, 3] | 2 [2, 3] | 4 [2, 6] |
|  | ≥3 per pregnancy | 14’094 | 12’092 | 1’566 | 359 | 77 |
|  | ≥4 per pregnancy | 6’113 | 5’198 | 671 | 184 | 60 |
|  | ≥5 per pregnancy | 2’953 | 2’508 | 303 | 100 | 42 |
|  | ≥6 per pregnancy | 1’643 | 1’384 | 175 | 53 | 31 |
| Metabolic causes of CKD |  |  |  |  |  |  |
| BMI [kg/m^2^]^g^ | Underweight (<18.5) | 1’118 (3.1) | 1’008 (3.3) | 94 (2.2) | 15 (1.7) | NR |
|  | Normal weight (18.5 to <24) | 12’089 (34.0) | 10’257 (33.8) | 1’484 (34.6) | 304 (35.4) | 44 (37.6) |
|  | Overweight (25 to <30) | 7’038 (19.8) | 6’025 (19.9) | 824 (19.2) | 170 (19.8) | 19 (16.2) |
|  | Obesity  (≥30) | 6’832 (19.2) | 5’795 (19.1) | 840 (19.6) | 174 (20.3) | 23 (19.7) |
|  | Unknown | 8’517 (23.9) | 7’239 (23.9) | 1’053 (24.5) | 195 (22.7) | 30 (25.6) |
| DM^h, i^ |  | 3’544 (10.0) | 3’035 (10.0) | 414 (9.6) | 79 (9.2) | 16 (13.7) |
| HT^h, j^ |  | 6’952 (19.5) | 5’759 (19.0) | 924 (21.5) | 221 (25.8) | 48 (41.0) |
| DM^h^ and/or overweight/obesity^g^ |  | 15’219 (42.8) | 12’961 (42.7) | 1’834 (42.7) | 373 (43.5) | 51 (43.6) |
| DM^h, i^ and/or HT^h, j^ and/or overweight/obesity^g^ |  | 18’645 (52.4) | 15’820 (52.2) | 2’278 (53.0) | 473 (55.1) | 74 (63.2) |
| Further potential causes of CKD |  |  |  |  |  |  |
| Kidney disease^k^ | Overall | 249 (0.7) | 0 (0.0) | 130 (3.0) | 58 (6.8) | 61 (52.1) |
|  | Autoimmune^l^ | 44 (0.1) | 0 (0.0) | 33 (0.8) | 9 (1.0) | NR |
|  | Non-immune-mediated^m^ | 58 (0.2) | 0 (0.0) | 27 (0.6) | 13 (1.5) | 18 (15.4) |
|  | Unspecified | 147 (0.4) | 0 (0.0) | 70 (1.6) | 36 (4.2) | 41 (35.0) |
| Other autoimmune disease (exclusive autoimmune kidney disease)^k, n^ |  | 679 (1.9) | 562 (1.9) | 88 (2.0) | 26 (3.0) | NR |
| Other potential cause^k, o^ |  | 203 (0.6) | 168 (0.6) | 28 (0.7) | NR | NR |

Values are the number (%) unless indicated otherwise.

**Abbreviations:**

CPRD = Clinical Practice Research Datalink, HES = Hospital Episode Statistics, CKD = chronic kidney disease, eGFR = estimated glomerular filtration rate, IQR = interquartile range, SCr = serum creatinine, BMI = body mass index, DM = diabetes mellitus type 1 and 2, HT = hypertension, Dx = diagnosis, Rx = prescription, HbA1c = hemoglobin A1c, NR = not reported (because cell sizes <5 patients)

**Footnote:**

^a^ baseline = (pregnancy start date-365 days) until pregnancy start date

^b^ trimester 1 = pregnancy start date until (pregnancy start date+93.3 days)

^c^ Conversion factor for µmol/L: multiplication by 88.4

^d^ Code for current smoker between start of baseline^a^ and delivery date

^e^ Read code for ‘current’ and ≥14 alcohol units per week (average of units measured any time before end of trimester 1)

^f^ observation period = (pregnancy start date-365 days) until (delivery date+365 days)

^g^ Last recording within three years before trimester 1 (classification according to Centers for Disease Control and Prevention^6^)

^h^ Last recording any time before end of trimester 1^b^

^i^ Dx and/or Rx and/or HbA1c level ≥6.5%

^j^ Dx and/or Rx

^k^ Last recording any time prior to the delivery date

^l^ Chronic glomerulonephritis and/or vasculitis (small and medium vessel vasculitis, i.e. polyarteritis nodosa, microscopic polyangiitis, Kawasaki disease, Churg-Strauss disease, Wegener’s granulomatosis, arteritis not otherwise specified, Henoch-Schonlein purpura, hypersensitivity angiitis) and/or hemolytic uremic syndrome and/or other miscellaneous immune-mediated kidney disease

^m^ Cystic kidney disease and/or other miscellaneous non-immune-mediated kidney disease

^n^ Systemic lupus erythematosus and/or rheumatoid arthritis or other inflammatory polyarthropathies and/or sjögren syndrome or sicca syndrome and/or dermatomyositis or polymyositis and/or systemic sclerosis and/or other connective tissue disease (incl. polymyalgia rheumatica, eosinophilia myalgia))

^o^ Amyloidosis and/or gout and/or chronic liver disease

## Changes in SCr levels in CPRD GOLD population

**Figure S2:** CPRD GOLD population: Median SCr levels (IQR) [mg/dL*] during baseline (before week 0), pregnancy (weeks 0-40), and postpartum period (after week 40). Changes in SCr levels are presented as two-week period medians among pregnancies categorized as G1 (eGFR≥90 ml/min/1.73 m2), G2-high (eGFR=75-89 ml/min/1.73 m²), or G2-low (eGFR=60-74 ml/min/1.73 m²) at baseline, and as ten-week period medians among pregnancies categorized as G3/G4 (eGFR=15-59 ml/min/1.73 m²). Median SCr levels are placed in the center of each time period. *Conversion factor for µmol/L: multiplication by 88.4


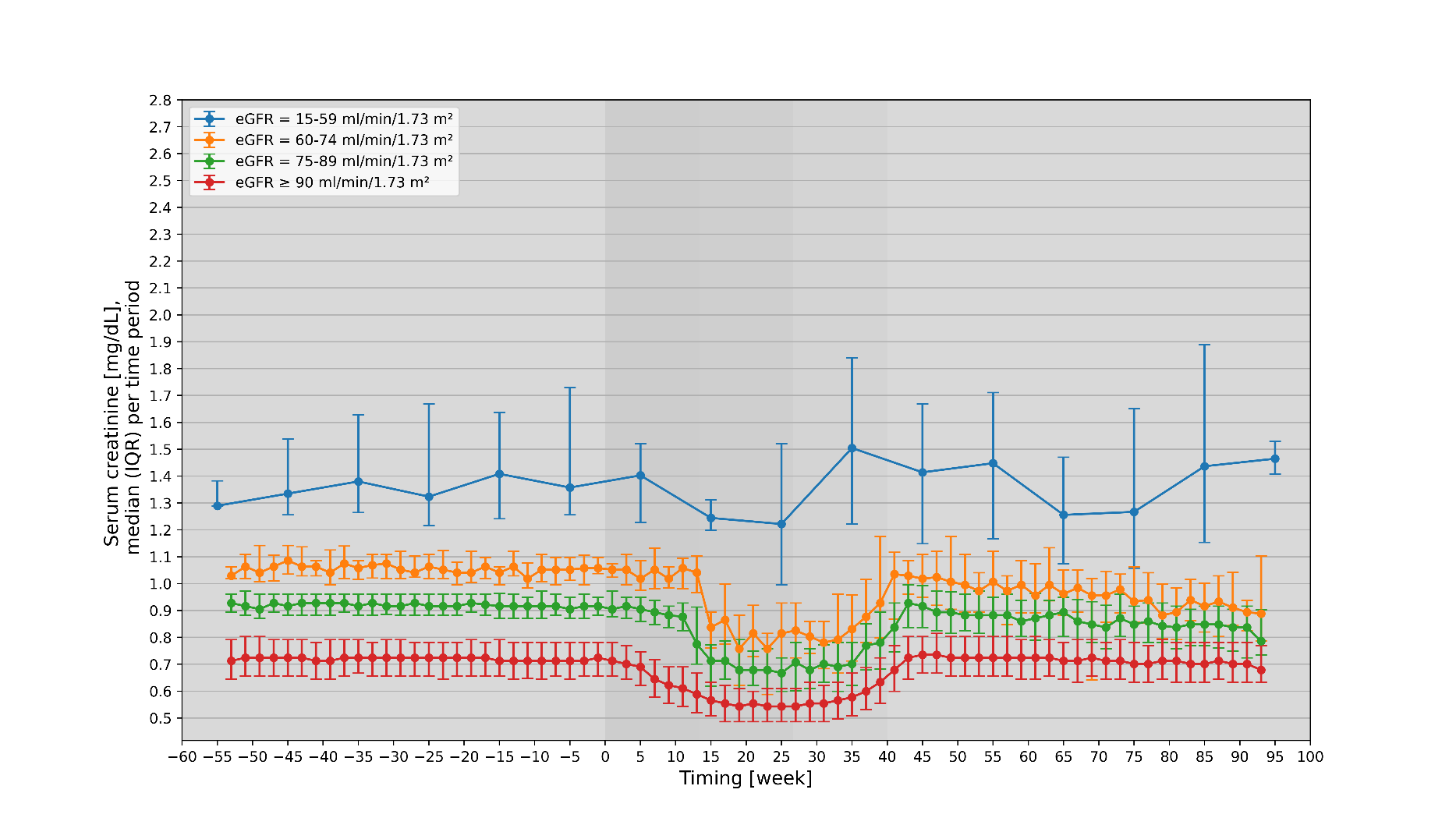


Abbreviations: SCr = serum creatinine, IQR = interquartile range, CPRD = Clinical Practice Research Datalink, eGFR = estimated glomerular filtration rate

We observed more variability in SCr levels in pregnancies categorized as G3/G4 at baseline (eGFR=15-59 ml/min/1.73 m^2^) before restriction to pregnancies with recorded GAB in linked HES-APC data (final (linked) study population). This highlights the importance of correctly defining pregnancy windows in populations with high proportions of preterm delivery.

## Numeric information of Figure S2 (changes in SCr over time) in CPRD GOLD population (not used for final analyses)

**Table S6:** CPRD GOLD population: Median SCr levels [mg/dL*] with percentiles and number of pregnancies in each time period (i.e., between period start and period end) by presence and severity of reduced baseline eGFR (cat = 1: G3/G4 (eGFR=15-59 ml/min/1.73 m²), cat = 2: G2-low (eGFR=60-74 ml/min/1.73 m²), cat = 3: G2-high (eGFR=75-89 ml/min/1.73 m²), cat = 4: G1 (eGFR≥ 90 ml/min/1.73 m2)). *Conversion factor for µmol/L: multiplication by 88.4

| **cat** | **start_ day** | **end_**  **day** | **start_**  **week** | **end_**  **week** | **n_**  **preg** | **n_**  **scr** | **scr_**  **median** | **p0** | **p25** | **p75** | **p100** |
| --- | --- | --- | --- | --- | --- | --- | --- | --- | --- | --- | --- |
| 1 | -420 | -350 | -60 | -50 | 6 | 6 | 1.29 | 1.23 | 1.29 | 1.38 | 1.80 |
| 1 | -350 | -280 | -50 | -40 | 28 | 33 | 1.33 | 1.17 | 1.26 | 1.54 | 1.95 |
| 1 | -280 | -210 | -40 | -30 | 21 | 30 | 1.38 | 0.97 | 1.26 | 1.63 | 2.59 |
| 1 | -210 | -140 | -30 | -20 | 30 | 39 | 1.32 | 1.07 | 1.22 | 1.67 | 2.22 |
| 1 | -140 | -70 | -20 | -10 | 33 | 40 | 1.41 | 1.09 | 1.24 | 1.64 | 3.35 |
| 1 | -70 | 0 | -10 | 0 | 32 | 37 | 1.36 | 1.02 | 1.26 | 1.73 | 3.39 |
| 1 | 0 | 70 | 0 | 10 | 35 | 43 | 1.40 | 1.05 | 1.23 | 1.52 | 2.22 |
| 1 | 70 | 140 | 10 | 20 | 28 | 33 | 1.24 | 0.42 | 1.20 | 1.31 | 2.04 |
| 1 | 140 | 210 | 20 | 30 | 30 | 43 | 1.22 | 0.81 | 1.00 | 1.52 | 2.29 |
| 1 | 210 | 280 | 30 | 40 | 34 | 62 | 1.50 | 0.74 | 1.22 | 1.84 | 3.40 |
| 1 | 280 | 350 | 40 | 50 | 40 | 47 | 1.41 | 0.72 | 1.15 | 1.67 | 2.84 |
| 1 | 350 | 420 | 50 | 60 | 30 | 34 | 1.45 | 0.83 | 1.17 | 1.71 | 2.65 |
| 1 | 420 | 490 | 60 | 70 | 21 | 23 | 1.26 | 0.85 | 1.07 | 1.47 | 3.29 |
| 1 | 490 | 560 | 70 | 80 | 19 | 23 | 1.27 | 0.79 | 1.06 | 1.65 | 3.01 |
| 1 | 560 | 630 | 80 | 90 | 22 | 25 | 1.44 | 0.81 | 1.15 | 1.89 | 3.17 |
| 1 | 630 | 700 | 90 | 100 | 4 | 4 | 1.46 | 1.26 | 1.41 | 1.53 | 1.71 |
| 2 | -378 | -364 | -54 | -52 | 9 | 9 | 1.03 | 0.94 | 1.02 | 1.06 | 1.19 |
| 2 | -364 | -350 | -52 | -50 | 40 | 41 | 1.06 | 0.90 | 1.02 | 1.11 | 1.28 |
| 2 | -350 | -336 | -50 | -48 | 33 | 33 | 1.04 | 0.98 | 1.01 | 1.14 | 1.37 |
| 2 | -336 | -322 | -48 | -46 | 38 | 38 | 1.06 | 0.94 | 1.01 | 1.11 | 1.55 |
| 2 | -322 | -308 | -46 | -44 | 37 | 39 | 1.09 | 0.90 | 1.04 | 1.14 | 1.38 |
| 2 | -308 | -294 | -44 | -42 | 34 | 35 | 1.06 | 0.89 | 1.03 | 1.14 | 1.23 |
| 2 | -294 | -280 | -42 | -40 | 25 | 25 | 1.06 | 0.98 | 1.03 | 1.09 | 1.20 |
| 2 | -280 | -266 | -40 | -38 | 40 | 43 | 1.04 | 0.93 | 1.00 | 1.13 | 1.43 |
| 2 | -266 | -252 | -38 | -36 | 39 | 42 | 1.07 | 0.78 | 1.02 | 1.14 | 1.31 |
| 2 | -252 | -238 | -36 | -34 | 47 | 48 | 1.06 | 0.89 | 1.02 | 1.09 | 1.26 |
| 2 | -238 | -224 | -34 | -32 | 42 | 42 | 1.07 | 0.96 | 1.02 | 1.11 | 1.27 |
| 2 | -224 | -210 | -32 | -30 | 44 | 45 | 1.07 | 0.87 | 1.02 | 1.11 | 1.35 |
| 2 | -210 | -196 | -30 | -28 | 44 | 45 | 1.05 | 0.96 | 1.02 | 1.12 | 1.35 |
| 2 | -196 | -182 | -28 | -26 | 38 | 39 | 1.04 | 0.96 | 1.02 | 1.10 | 1.21 |
| 2 | -182 | -168 | -26 | -24 | 39 | 41 | 1.06 | 0.92 | 1.02 | 1.11 | 1.22 |
| 2 | -168 | -154 | -24 | -22 | 50 | 52 | 1.05 | 0.92 | 1.02 | 1.12 | 1.26 |
| 2 | -154 | -140 | -22 | -20 | 50 | 51 | 1.04 | 0.89 | 1.00 | 1.08 | 1.41 |
| 2 | -140 | -126 | -20 | -18 | 43 | 44 | 1.04 | 0.85 | 1.00 | 1.12 | 1.29 |
| 2 | -126 | -112 | -18 | -16 | 40 | 41 | 1.06 | 0.90 | 1.02 | 1.10 | 1.23 |
| 2 | -112 | -98 | -16 | -14 | 47 | 50 | 1.04 | 0.86 | 1.00 | 1.06 | 1.47 |
| 2 | -98 | -84 | -14 | -12 | 44 | 45 | 1.06 | 0.86 | 1.03 | 1.12 | 1.29 |
| 2 | -84 | -70 | -12 | -10 | 43 | 44 | 1.02 | 0.89 | 0.98 | 1.08 | 1.28 |
| 2 | -70 | -56 | -10 | -8 | 48 | 49 | 1.05 | 0.93 | 1.01 | 1.11 | 1.36 |
| 2 | -56 | -42 | -8 | -6 | 36 | 37 | 1.05 | 0.88 | 1.00 | 1.10 | 1.22 |
| 2 | -42 | -28 | -6 | -4 | 44 | 47 | 1.05 | 0.84 | 1.01 | 1.10 | 1.23 |
| 2 | -28 | -14 | -4 | -2 | 34 | 34 | 1.06 | 0.86 | 1.00 | 1.11 | 1.20 |
| 2 | -14 | 0 | -2 | 0 | 36 | 40 | 1.06 | 0.88 | 1.04 | 1.10 | 1.30 |
| 2 | 0 | 14 | 0 | 2 | 42 | 43 | 1.05 | 0.93 | 1.02 | 1.07 | 1.31 |
| 2 | 14 | 28 | 2 | 4 | 38 | 39 | 1.05 | 0.86 | 1.00 | 1.11 | 1.23 |
| 2 | 28 | 42 | 4 | 6 | 42 | 46 | 1.02 | 0.81 | 0.98 | 1.09 | 1.41 |
| 2 | 42 | 56 | 6 | 8 | 20 | 20 | 1.05 | 0.85 | 0.98 | 1.13 | 1.20 |
| 2 | 56 | 70 | 8 | 10 | 27 | 27 | 1.02 | 0.81 | 0.98 | 1.06 | 1.27 |
| 2 | 70 | 84 | 10 | 12 | 26 | 26 | 1.06 | 0.81 | 0.98 | 1.09 | 1.36 |
| 2 | 84 | 98 | 12 | 14 | 29 | 31 | 1.04 | 0.48 | 0.97 | 1.10 | 1.30 |
| 2 | 98 | 112 | 14 | 16 | 26 | 28 | 0.84 | 0.60 | 0.76 | 0.89 | 1.44 |
| 2 | 112 | 126 | 16 | 18 | 23 | 24 | 0.87 | 0.63 | 0.77 | 1.00 | 1.43 |
| 2 | 126 | 140 | 18 | 20 | 24 | 25 | 0.76 | 0.51 | 0.62 | 0.88 | 1.21 |
| 2 | 140 | 154 | 20 | 22 | 28 | 28 | 0.81 | 0.52 | 0.73 | 0.92 | 1.15 |
| 2 | 154 | 168 | 22 | 24 | 17 | 17 | 0.76 | 0.44 | 0.59 | 0.81 | 1.09 |
| 2 | 168 | 182 | 24 | 26 | 31 | 33 | 0.81 | 0.51 | 0.72 | 0.93 | 1.13 |
| 2 | 182 | 196 | 26 | 28 | 37 | 37 | 0.83 | 0.52 | 0.72 | 0.93 | 1.22 |
| 2 | 196 | 210 | 28 | 30 | 26 | 27 | 0.80 | 0.51 | 0.74 | 0.86 | 1.43 |
| 2 | 210 | 224 | 30 | 32 | 23 | 23 | 0.78 | 0.54 | 0.68 | 0.86 | 1.12 |
| 2 | 224 | 238 | 32 | 34 | 45 | 51 | 0.79 | 0.50 | 0.67 | 0.96 | 1.58 |
| 2 | 238 | 252 | 34 | 36 | 39 | 46 | 0.83 | 0.45 | 0.71 | 0.96 | 1.62 |
| 2 | 252 | 266 | 36 | 38 | 44 | 46 | 0.88 | 0.49 | 0.78 | 1.02 | 3.56 |
| 2 | 266 | 280 | 38 | 40 | 42 | 51 | 0.93 | 0.59 | 0.80 | 1.18 | 1.98 |
| 2 | 280 | 294 | 40 | 42 | 25 | 30 | 1.04 | 0.53 | 0.87 | 1.12 | 1.89 |
| 2 | 294 | 308 | 42 | 44 | 31 | 31 | 1.03 | 0.70 | 0.96 | 1.09 | 1.65 |
| 2 | 308 | 322 | 44 | 46 | 33 | 33 | 1.02 | 0.74 | 0.95 | 1.11 | 1.52 |
| 2 | 322 | 336 | 46 | 48 | 33 | 34 | 1.02 | 0.74 | 0.92 | 1.12 | 1.45 |
| 2 | 336 | 350 | 48 | 50 | 31 | 33 | 1.01 | 0.52 | 0.90 | 1.18 | 1.70 |
| 2 | 350 | 364 | 50 | 52 | 24 | 25 | 1.00 | 0.62 | 0.89 | 1.11 | 1.47 |
| 2 | 364 | 378 | 52 | 54 | 30 | 31 | 0.97 | 0.70 | 0.89 | 1.04 | 5.77 |
| 2 | 378 | 392 | 54 | 56 | 32 | 33 | 1.01 | 0.71 | 0.88 | 1.12 | 1.45 |
| 2 | 392 | 406 | 56 | 58 | 24 | 26 | 0.97 | 0.71 | 0.85 | 1.03 | 1.36 |
| 2 | 406 | 420 | 58 | 60 | 37 | 38 | 1.00 | 0.64 | 0.89 | 1.09 | 1.44 |
| 2 | 420 | 434 | 60 | 62 | 28 | 28 | 0.96 | 0.70 | 0.89 | 1.07 | 1.19 |
| 2 | 434 | 448 | 62 | 64 | 28 | 28 | 1.00 | 0.70 | 0.94 | 1.13 | 1.66 |
| 2 | 448 | 462 | 64 | 66 | 37 | 37 | 0.96 | 0.70 | 0.88 | 1.05 | 1.56 |
| 2 | 462 | 476 | 66 | 68 | 27 | 27 | 0.98 | 0.71 | 0.89 | 1.05 | 1.19 |
| 2 | 476 | 490 | 68 | 70 | 16 | 18 | 0.96 | 0.02 | 0.64 | 1.02 | 1.43 |
| 2 | 490 | 504 | 70 | 72 | 38 | 38 | 0.96 | 0.66 | 0.86 | 1.05 | 1.19 |
| 2 | 504 | 518 | 72 | 74 | 32 | 32 | 0.98 | 0.62 | 0.89 | 1.04 | 1.28 |
| 2 | 518 | 532 | 74 | 76 | 35 | 36 | 0.93 | 0.67 | 0.86 | 1.06 | 1.23 |
| 2 | 532 | 546 | 76 | 78 | 31 | 31 | 0.94 | 0.69 | 0.85 | 1.04 | 1.14 |
| 2 | 546 | 560 | 78 | 80 | 33 | 34 | 0.88 | 0.60 | 0.79 | 1.00 | 1.30 |
| 2 | 560 | 574 | 80 | 82 | 29 | 29 | 0.89 | 0.60 | 0.79 | 0.98 | 1.27 |
| 2 | 574 | 588 | 82 | 84 | 30 | 30 | 0.94 | 0.67 | 0.86 | 1.02 | 1.90 |
| 2 | 588 | 602 | 84 | 86 | 23 | 23 | 0.92 | 0.64 | 0.82 | 1.00 | 1.13 |
| 2 | 602 | 616 | 86 | 88 | 25 | 26 | 0.93 | 0.60 | 0.80 | 1.03 | 1.26 |
| 2 | 616 | 630 | 88 | 90 | 24 | 24 | 0.91 | 0.64 | 0.85 | 1.04 | 1.31 |
| 2 | 630 | 644 | 90 | 92 | 24 | 24 | 0.89 | 0.61 | 0.83 | 0.94 | 1.29 |
| 2 | 644 | 658 | 92 | 94 | 4 | 4 | 0.89 | 0.77 | 0.79 | 1.10 | 1.46 |
| 3 | -378 | -364 | -54 | -52 | 34 | 34 | 0.93 | 0.76 | 0.89 | 0.96 | 1.03 |
| 3 | -364 | -350 | -52 | -50 | 176 | 186 | 0.92 | 0.74 | 0.88 | 0.97 | 1.41 |
| 3 | -350 | -336 | -50 | -48 | 199 | 208 | 0.90 | 0.69 | 0.87 | 0.96 | 1.15 |
| 3 | -336 | -322 | -48 | -46 | 193 | 195 | 0.93 | 0.68 | 0.89 | 0.96 | 1.46 |
| 3 | -322 | -308 | -46 | -44 | 209 | 215 | 0.92 | 0.76 | 0.88 | 0.96 | 1.33 |
| 3 | -308 | -294 | -44 | -42 | 194 | 197 | 0.93 | 0.72 | 0.88 | 0.96 | 1.21 |
| 3 | -294 | -280 | -42 | -40 | 188 | 193 | 0.93 | 0.76 | 0.88 | 0.96 | 1.22 |
| 3 | -280 | -266 | -40 | -38 | 221 | 225 | 0.93 | 0.76 | 0.88 | 0.96 | 1.16 |
| 3 | -266 | -252 | -38 | -36 | 192 | 198 | 0.93 | 0.77 | 0.89 | 0.96 | 1.12 |
| 3 | -252 | -238 | -36 | -34 | 196 | 200 | 0.92 | 0.69 | 0.88 | 0.96 | 1.41 |
| 3 | -238 | -224 | -34 | -32 | 208 | 211 | 0.93 | 0.74 | 0.88 | 0.96 | 1.22 |
| 3 | -224 | -210 | -32 | -30 | 202 | 202 | 0.92 | 0.80 | 0.89 | 0.96 | 1.27 |
| 3 | -210 | -196 | -30 | -28 | 215 | 220 | 0.92 | 0.74 | 0.88 | 0.96 | 1.17 |
| 3 | -196 | -182 | -28 | -26 | 214 | 219 | 0.93 | 0.78 | 0.88 | 0.96 | 1.49 |
| 3 | -182 | -168 | -26 | -24 | 189 | 191 | 0.92 | 0.75 | 0.88 | 0.96 | 1.11 |
| 3 | -168 | -154 | -24 | -22 | 195 | 196 | 0.92 | 0.77 | 0.88 | 0.96 | 1.27 |
| 3 | -154 | -140 | -22 | -20 | 208 | 213 | 0.92 | 0.71 | 0.88 | 0.96 | 1.29 |
| 3 | -140 | -126 | -20 | -18 | 193 | 193 | 0.93 | 0.69 | 0.89 | 0.96 | 1.19 |
| 3 | -126 | -112 | -18 | -16 | 208 | 212 | 0.92 | 0.78 | 0.88 | 0.96 | 1.13 |
| 3 | -112 | -98 | -16 | -14 | 208 | 212 | 0.92 | 0.70 | 0.87 | 0.96 | 1.36 |
| 3 | -98 | -84 | -14 | -12 | 202 | 209 | 0.92 | 0.67 | 0.87 | 0.96 | 1.24 |
| 3 | -84 | -70 | -12 | -10 | 207 | 215 | 0.92 | 0.75 | 0.87 | 0.95 | 1.23 |
| 3 | -70 | -56 | -10 | -8 | 215 | 220 | 0.92 | 0.76 | 0.87 | 0.97 | 1.22 |
| 3 | -56 | -42 | -8 | -6 | 229 | 233 | 0.92 | 0.68 | 0.88 | 0.96 | 1.47 |
| 3 | -42 | -28 | -6 | -4 | 209 | 210 | 0.90 | 0.71 | 0.87 | 0.95 | 1.21 |
| 3 | -28 | -14 | -4 | -2 | 223 | 229 | 0.92 | 0.70 | 0.87 | 0.96 | 1.19 |
| 3 | -14 | 0 | -2 | 0 | 205 | 212 | 0.92 | 0.75 | 0.88 | 0.95 | 1.36 |
| 3 | 0 | 14 | 0 | 2 | 203 | 207 | 0.90 | 0.71 | 0.88 | 0.97 | 1.12 |
| 3 | 14 | 28 | 2 | 4 | 220 | 221 | 0.92 | 0.77 | 0.87 | 0.95 | 1.18 |
| 3 | 28 | 42 | 4 | 6 | 153 | 154 | 0.90 | 0.74 | 0.86 | 0.95 | 1.53 |
| 3 | 42 | 56 | 6 | 8 | 123 | 127 | 0.89 | 0.70 | 0.85 | 0.94 | 1.12 |
| 3 | 56 | 70 | 8 | 10 | 134 | 139 | 0.88 | 0.62 | 0.84 | 0.92 | 1.03 |
| 3 | 70 | 84 | 10 | 12 | 107 | 110 | 0.88 | 0.52 | 0.83 | 0.93 | 1.56 |
| 3 | 84 | 98 | 12 | 14 | 82 | 82 | 0.77 | 0.46 | 0.70 | 0.91 | 1.05 |
| 3 | 98 | 112 | 14 | 16 | 114 | 116 | 0.71 | 0.50 | 0.62 | 0.77 | 1.46 |
| 3 | 112 | 126 | 16 | 18 | 113 | 115 | 0.71 | 0.51 | 0.64 | 0.79 | 1.26 |
| 3 | 126 | 140 | 18 | 20 | 92 | 93 | 0.68 | 0.45 | 0.61 | 0.79 | 1.01 |
| 3 | 140 | 154 | 20 | 22 | 97 | 100 | 0.68 | 0.48 | 0.62 | 0.75 | 1.04 |
| 3 | 154 | 168 | 22 | 24 | 106 | 107 | 0.68 | 0.07 | 0.61 | 0.76 | 1.11 |
| 3 | 168 | 182 | 24 | 26 | 107 | 109 | 0.67 | 0.48 | 0.60 | 0.72 | 1.03 |
| 3 | 182 | 196 | 26 | 28 | 121 | 122 | 0.71 | 0.45 | 0.60 | 0.78 | 1.02 |
| 3 | 196 | 210 | 28 | 30 | 128 | 135 | 0.68 | 0.06 | 0.61 | 0.76 | 1.00 |
| 3 | 210 | 224 | 30 | 32 | 120 | 124 | 0.70 | 0.43 | 0.63 | 0.77 | 1.45 |
| 3 | 224 | 238 | 32 | 34 | 135 | 140 | 0.69 | 0.38 | 0.60 | 0.78 | 1.01 |
| 3 | 238 | 252 | 34 | 36 | 137 | 146 | 0.70 | 0.33 | 0.62 | 0.78 | 1.06 |
| 3 | 252 | 266 | 36 | 38 | 149 | 164 | 0.77 | 0.48 | 0.68 | 0.85 | 2.57 |
| 3 | 266 | 280 | 38 | 40 | 140 | 154 | 0.78 | 0.51 | 0.68 | 0.89 | 1.73 |
| 3 | 280 | 294 | 40 | 42 | 113 | 121 | 0.84 | 0.42 | 0.75 | 0.93 | 2.49 |
| 3 | 294 | 308 | 42 | 44 | 127 | 132 | 0.93 | 0.58 | 0.84 | 1.00 | 1.53 |
| 3 | 308 | 322 | 44 | 46 | 148 | 150 | 0.92 | 0.60 | 0.84 | 0.99 | 2.49 |
| 3 | 322 | 336 | 46 | 48 | 178 | 183 | 0.89 | 0.02 | 0.83 | 0.97 | 1.44 |
| 3 | 336 | 350 | 48 | 50 | 137 | 142 | 0.89 | 0.62 | 0.81 | 0.97 | 1.24 |
| 3 | 350 | 364 | 50 | 52 | 126 | 127 | 0.88 | 0.53 | 0.83 | 0.96 | 1.20 |
| 3 | 364 | 378 | 52 | 54 | 116 | 118 | 0.88 | 0.40 | 0.81 | 0.97 | 3.01 |
| 3 | 378 | 392 | 54 | 56 | 167 | 171 | 0.88 | 0.48 | 0.80 | 0.95 | 1.14 |
| 3 | 392 | 406 | 56 | 58 | 147 | 147 | 0.88 | 0.54 | 0.80 | 0.96 | 1.14 |
| 3 | 406 | 420 | 58 | 60 | 130 | 131 | 0.86 | 0.64 | 0.80 | 0.94 | 1.15 |
| 3 | 420 | 434 | 60 | 62 | 172 | 172 | 0.87 | 0.52 | 0.79 | 0.96 | 1.56 |
| 3 | 434 | 448 | 62 | 64 | 125 | 127 | 0.88 | 0.35 | 0.79 | 0.94 | 1.28 |
| 3 | 448 | 462 | 64 | 66 | 133 | 135 | 0.89 | 0.52 | 0.80 | 0.95 | 1.63 |
| 3 | 462 | 476 | 66 | 68 | 153 | 156 | 0.86 | 0.46 | 0.79 | 0.94 | 1.46 |
| 3 | 476 | 490 | 68 | 70 | 134 | 135 | 0.85 | 0.50 | 0.78 | 0.93 | 1.36 |
| 3 | 490 | 504 | 70 | 72 | 106 | 108 | 0.84 | 0.08 | 0.76 | 0.92 | 1.18 |
| 3 | 504 | 518 | 72 | 74 | 115 | 117 | 0.87 | 0.50 | 0.80 | 0.96 | 1.19 |
| 3 | 518 | 532 | 74 | 76 | 133 | 135 | 0.85 | 0.59 | 0.78 | 0.92 | 1.32 |
| 3 | 532 | 546 | 76 | 78 | 132 | 135 | 0.86 | 0.52 | 0.78 | 0.93 | 1.14 |
| 3 | 546 | 560 | 78 | 80 | 130 | 134 | 0.84 | 0.50 | 0.78 | 0.93 | 1.18 |
| 3 | 560 | 574 | 80 | 82 | 138 | 140 | 0.84 | 0.53 | 0.76 | 0.92 | 1.31 |
| 3 | 574 | 588 | 82 | 84 | 104 | 108 | 0.85 | 0.46 | 0.77 | 0.90 | 1.37 |
| 3 | 588 | 602 | 84 | 86 | 130 | 134 | 0.85 | 0.51 | 0.77 | 0.92 | 1.11 |
| 3 | 602 | 616 | 86 | 88 | 124 | 126 | 0.85 | 0.55 | 0.76 | 0.92 | 6.81 |
| 3 | 616 | 630 | 88 | 90 | 96 | 98 | 0.84 | 0.55 | 0.75 | 0.90 | 1.12 |
| 3 | 630 | 644 | 90 | 92 | 100 | 101 | 0.84 | 0.43 | 0.72 | 0.92 | 1.27 |
| 3 | 644 | 658 | 92 | 94 | 28 | 30 | 0.79 | 0.43 | 0.74 | 0.90 | 1.13 |
| 4 | -378 | -364 | -54 | -52 | 226 | 228 | 0.71 | 0.45 | 0.64 | 0.79 | 1.09 |
| 4 | -364 | -350 | -52 | -50 | 1282 | 1304 | 0.72 | 0.31 | 0.66 | 0.80 | 1.04 |
| 4 | -350 | -336 | -50 | -48 | 1320 | 1340 | 0.72 | 0.34 | 0.66 | 0.80 | 1.09 |
| 4 | -336 | -322 | -48 | -46 | 1259 | 1280 | 0.72 | 0.35 | 0.66 | 0.79 | 1.03 |
| 4 | -322 | -308 | -46 | -44 | 1319 | 1336 | 0.72 | 0.14 | 0.66 | 0.79 | 1.30 |
| 4 | -308 | -294 | -44 | -42 | 1281 | 1313 | 0.72 | 0.09 | 0.66 | 0.79 | 1.09 |
| 4 | -294 | -280 | -42 | -40 | 1323 | 1341 | 0.71 | 0.04 | 0.64 | 0.78 | 1.15 |
| 4 | -280 | -266 | -40 | -38 | 1342 | 1362 | 0.71 | 0.35 | 0.64 | 0.79 | 1.04 |
| 4 | -266 | -252 | -38 | -36 | 1383 | 1407 | 0.72 | 0.41 | 0.66 | 0.79 | 1.10 |
| 4 | -252 | -238 | -36 | -34 | 1341 | 1357 | 0.72 | 0.09 | 0.67 | 0.79 | 1.02 |
| 4 | -238 | -224 | -34 | -32 | 1395 | 1429 | 0.72 | 0.32 | 0.66 | 0.78 | 1.14 |
| 4 | -224 | -210 | -32 | -30 | 1309 | 1330 | 0.72 | 0.36 | 0.66 | 0.79 | 1.18 |
| 4 | -210 | -196 | -30 | -28 | 1386 | 1413 | 0.72 | 0.05 | 0.66 | 0.79 | 1.11 |
| 4 | -196 | -182 | -28 | -26 | 1358 | 1374 | 0.72 | 0.09 | 0.66 | 0.78 | 1.13 |
| 4 | -182 | -168 | -26 | -24 | 1417 | 1446 | 0.72 | 0.09 | 0.66 | 0.78 | 1.06 |
| 4 | -168 | -154 | -24 | -22 | 1382 | 1403 | 0.72 | 0.38 | 0.64 | 0.78 | 1.49 |
| 4 | -154 | -140 | -22 | -20 | 1352 | 1376 | 0.72 | 0.23 | 0.66 | 0.78 | 1.09 |
| 4 | -140 | -126 | -20 | -18 | 1412 | 1439 | 0.72 | 0.36 | 0.66 | 0.79 | 1.02 |
| 4 | -126 | -112 | -18 | -16 | 1389 | 1419 | 0.72 | 0.42 | 0.66 | 0.78 | 1.09 |
| 4 | -112 | -98 | -16 | -14 | 1482 | 1514 | 0.71 | 0.07 | 0.66 | 0.79 | 1.14 |
| 4 | -98 | -84 | -14 | -12 | 1417 | 1436 | 0.71 | 0.20 | 0.64 | 0.78 | 1.09 |
| 4 | -84 | -70 | -12 | -10 | 1453 | 1478 | 0.71 | 0.33 | 0.65 | 0.78 | 1.22 |
| 4 | -70 | -56 | -10 | -8 | 1495 | 1518 | 0.71 | 0.10 | 0.64 | 0.78 | 1.13 |
| 4 | -56 | -42 | -8 | -6 | 1470 | 1489 | 0.71 | 0.37 | 0.66 | 0.78 | 1.20 |
| 4 | -42 | -28 | -6 | -4 | 1534 | 1558 | 0.71 | 0.09 | 0.64 | 0.78 | 1.13 |
| 4 | -28 | -14 | -4 | -2 | 1536 | 1558 | 0.71 | 0.09 | 0.66 | 0.78 | 1.30 |
| 4 | -14 | 0 | -2 | 0 | 1542 | 1561 | 0.72 | 0.33 | 0.66 | 0.78 | 0.98 |
| 4 | 0 | 14 | 0 | 2 | 1512 | 1525 | 0.71 | 0.26 | 0.66 | 0.78 | 1.12 |
| 4 | 14 | 28 | 2 | 4 | 1519 | 1543 | 0.70 | 0.05 | 0.64 | 0.77 | 1.11 |
| 4 | 28 | 42 | 4 | 6 | 1691 | 1722 | 0.69 | 0.03 | 0.62 | 0.75 | 1.20 |
| 4 | 42 | 56 | 6 | 8 | 1906 | 1959 | 0.64 | 0.09 | 0.58 | 0.72 | 1.33 |
| 4 | 56 | 70 | 8 | 10 | 1921 | 1972 | 0.62 | 0.11 | 0.55 | 0.69 | 0.97 |
| 4 | 70 | 84 | 10 | 12 | 1587 | 1627 | 0.61 | 0.06 | 0.54 | 0.69 | 0.94 |
| 4 | 84 | 98 | 12 | 14 | 1148 | 1202 | 0.59 | 0.06 | 0.52 | 0.67 | 1.14 |
| 4 | 98 | 112 | 14 | 16 | 781 | 794 | 0.57 | 0.28 | 0.51 | 0.63 | 1.03 |
| 4 | 112 | 126 | 16 | 18 | 784 | 805 | 0.55 | 0.24 | 0.49 | 0.62 | 1.01 |
| 4 | 126 | 140 | 18 | 20 | 806 | 818 | 0.54 | 0.28 | 0.49 | 0.61 | 1.74 |
| 4 | 140 | 154 | 20 | 22 | 716 | 734 | 0.55 | 0.29 | 0.49 | 0.60 | 0.98 |
| 4 | 154 | 168 | 22 | 24 | 802 | 823 | 0.54 | 0.04 | 0.49 | 0.61 | 0.88 |
| 4 | 168 | 182 | 24 | 26 | 894 | 928 | 0.54 | 0.32 | 0.49 | 0.61 | 1.31 |
| 4 | 182 | 196 | 26 | 28 | 880 | 901 | 0.54 | 0.03 | 0.49 | 0.61 | 1.20 |
| 4 | 196 | 210 | 28 | 30 | 856 | 887 | 0.55 | 0.27 | 0.49 | 0.63 | 1.35 |
| 4 | 210 | 224 | 30 | 32 | 828 | 862 | 0.55 | 0.04 | 0.49 | 0.62 | 1.32 |
| 4 | 224 | 238 | 32 | 34 | 874 | 919 | 0.57 | 0.31 | 0.50 | 0.63 | 1.32 |
| 4 | 238 | 252 | 34 | 36 | 918 | 979 | 0.58 | 0.28 | 0.51 | 0.67 | 1.57 |
| 4 | 252 | 266 | 36 | 38 | 926 | 1030 | 0.60 | 0.28 | 0.53 | 0.69 | 1.53 |
| 4 | 266 | 280 | 38 | 40 | 906 | 1058 | 0.63 | 0.31 | 0.55 | 0.72 | 1.55 |
| 4 | 280 | 294 | 40 | 42 | 924 | 1038 | 0.68 | 0.05 | 0.60 | 0.77 | 1.88 |
| 4 | 294 | 308 | 42 | 44 | 927 | 950 | 0.72 | 0.35 | 0.64 | 0.80 | 1.55 |
| 4 | 308 | 322 | 44 | 46 | 956 | 974 | 0.74 | 0.25 | 0.67 | 0.80 | 1.24 |
| 4 | 322 | 336 | 46 | 48 | 1200 | 1215 | 0.74 | 0.35 | 0.67 | 0.81 | 1.46 |
| 4 | 336 | 350 | 48 | 50 | 1097 | 1112 | 0.72 | 0.40 | 0.66 | 0.80 | 1.33 |
| 4 | 350 | 364 | 50 | 52 | 1021 | 1044 | 0.72 | 0.36 | 0.66 | 0.80 | 1.89 |
| 4 | 364 | 378 | 52 | 54 | 1024 | 1038 | 0.72 | 0.41 | 0.66 | 0.80 | 1.30 |
| 4 | 378 | 392 | 54 | 56 | 999 | 1014 | 0.72 | 0.07 | 0.66 | 0.80 | 1.65 |
| 4 | 392 | 406 | 56 | 58 | 1076 | 1089 | 0.72 | 0.07 | 0.66 | 0.80 | 1.23 |
| 4 | 406 | 420 | 58 | 60 | 1055 | 1073 | 0.72 | 0.31 | 0.64 | 0.79 | 1.22 |
| 4 | 420 | 434 | 60 | 62 | 1024 | 1044 | 0.72 | 0.33 | 0.64 | 0.80 | 1.35 |
| 4 | 434 | 448 | 62 | 64 | 1021 | 1030 | 0.72 | 0.25 | 0.66 | 0.79 | 1.71 |
| 4 | 448 | 462 | 64 | 66 | 987 | 1006 | 0.71 | 0.36 | 0.64 | 0.78 | 1.64 |
| 4 | 462 | 476 | 66 | 68 | 980 | 991 | 0.71 | 0.41 | 0.63 | 0.79 | 1.18 |
| 4 | 476 | 490 | 68 | 70 | 986 | 997 | 0.72 | 0.35 | 0.66 | 0.79 | 1.30 |
| 4 | 490 | 504 | 70 | 72 | 952 | 965 | 0.71 | 0.06 | 0.64 | 0.78 | 1.47 |
| 4 | 504 | 518 | 72 | 74 | 891 | 900 | 0.71 | 0.36 | 0.64 | 0.79 | 1.22 |
| 4 | 518 | 532 | 74 | 76 | 934 | 948 | 0.70 | 0.28 | 0.63 | 0.78 | 1.90 |
| 4 | 532 | 546 | 76 | 78 | 883 | 892 | 0.70 | 0.36 | 0.63 | 0.77 | 1.52 |
| 4 | 546 | 560 | 78 | 80 | 881 | 898 | 0.71 | 0.06 | 0.64 | 0.79 | 2.06 |
| 4 | 560 | 574 | 80 | 82 | 891 | 908 | 0.71 | 0.36 | 0.63 | 0.78 | 1.57 |
| 4 | 574 | 588 | 82 | 84 | 820 | 832 | 0.70 | 0.27 | 0.63 | 0.78 | 1.49 |
| 4 | 588 | 602 | 84 | 86 | 797 | 812 | 0.70 | 0.43 | 0.63 | 0.78 | 1.33 |
| 4 | 602 | 616 | 86 | 88 | 895 | 912 | 0.71 | 0.36 | 0.64 | 0.78 | 1.55 |
| 4 | 616 | 630 | 88 | 90 | 791 | 803 | 0.70 | 0.35 | 0.63 | 0.78 | 1.24 |
| 4 | 630 | 644 | 90 | 92 | 671 | 681 | 0.70 | 0.36 | 0.63 | 0.78 | 1.10 |
| 4 | 644 | 658 | 92 | 94 | 148 | 148 | 0.68 | 0.40 | 0.63 | 0.77 | 0.98 |

Abbreviations: cat = category, start_day = day of time period start, end_day = day of time period end, start_week = week of time period start, end_week = week of time period end, n_preg = number of pregnancy in corresponding time period, n_scr = number of SCr measurements in corresponding time period, scr_median = median SCr levels in corresponding time period, p0 = minimum (SCr level), p25 = 25^th^ percentile (SCr level), p75 = 75^th^ percentile (SCr level), p100 = maximum (SCr level)

# **Further discussion**

## CPRD GOLD as unique data source for our research question

Overall, CPRD GOLD is a reliable data source to evaluate SCr measurements. The high frequency of SCr measurements in CPRD GOLD compared to other electronic databases is unique and mainly due to financial incentives and official guidelines for UK GPs to monitor and record eGFR in high-risk patients.^7–10^ The primary care nature of CPRD GOLD allowed us to include a representative population of patients with mildly reduced eGFR categorized as G2, which were under-represented in prior studies. Also, linkage of CPRD GOLD with HES-APC data allowed an accurate estimation of pregnancy start and delivery dates.

## Baseline characteristics

Metabolic disorders are common causes of CKD, which is reflected in our study population.^11–16^ Compared to the UK female population of childbearing age, diabetes and hypertension were approximately 5-times more common among those categorized as G2-high/G2-low and up to 12-times more common in those categorized as G3/G4.^17^ The higher prevalence of diabetes in pregnancies with normal or high eGFR (G1) in our study population (10.6%) is likely explained by the fact that patients with known risk factors for CKD are more likely to have SCr levels measured. On the other hand, although autoimmune diseases (predominantly glomerulonephritis^18–20^) are common causes of CKD in patients of childbearing age, a diagnosis of autoimmune kidney disease was recorded in only 0.4% and 0.5% of pregnancies categorized as G2-high or G2-low, and in 3.8% of those categorized as G3/G4. Again, this suggests, that patients with complex autoimmune causes of CKD may be more likely under specialist care, but poor diagnostic coding of CKD in CPRD GOLD has also been described previously and may likely also partly explain this low proportion.^21^

## Pregnancies categorized as G3/G4: Changes in SCr levels and representativeness of the G3/G4 category

Results for pregnancies categorized as G3/G4 have to be interpreted carefully due to small sample size and potential selection bias. Overall, SCr levels adapted to physiological changes in our study, but the relative increase in SCr levels towards the end of pregnancy was somewhat greater compared to those categorized as G1 or G2-high/G2-low and was followed by slightly but not drastically increased SCr levels postpartum (vs. pre-pregnancy) and with very large interquartile ranges. Case series from the 1980/90s reported that kidneys of 30-50% of patients with moderate-severe CKD recruited at US hospitals were not able to adapt to physiological changes during pregnancy.^22,23^ Furthermore, an Italian hospital-based study (1977-2004) reported a diminished renal adaptation among those with most severe CKD (eGFR<40 and proteinuria≥1g/day) when compared to patients with less severe CKD.^24^ Furthermore, in a study including 178 pregnancies from UK tertiary care centers, 46% of patients with a pre-pregnancy eGFR<60 (G3-G5) lost ≥25% of their eGFR (compared to pre-pregnancy levels) or required renal replacement therapy within one year postpartum.^25^ While such studies likely over-sampled cases with more severe renal impairment due to their focus on tertiary care, our study likely under-represents patients with more severely reduced renal filtration, because they are more likely under exclusive specialist care. It is important that future studies evaluate a representative group of patients with moderate-severe CKD and evaluate individual risk factors for insufficient renal adaptation during and after pregnancy, with focus on patients with eGFR=60-74 and eGFR<60, to identify those patients in need of closer medical surveillance during and after pregnancy.

On the other hand, previously reported increased risks of prematurity and underweight among patients with most severe CKD could be reproduced in our study, suggesting that our study population is not completely biased towards the less severe cases of CKD.^11,16,24,26,27^

## Materno-fetal outcomes

Among pregnancies with reduced eGFR=15-59 (G3/G4), 30.2% delivered prematurely and 22.6% of newborns were underweight. Contrarily, among pregnancies with reduced eGFR=60-74 (G2-low), the proportion of preterm deliveries (9.8%) or underweight newborns (7.7%) were only slightly higher compared to the overall UK population (7.6% and 7.0%, respectively), proposing that a baseline eGFR between 60 and 74 is not necessarily associated with an increased risk of prematurity and low birth weight.^28^

## Limitations

The following limitations need to be considered. First, we defined G-categories of renal filtration based on baseline eGFR. Albuminuria is under-recorded in CPRD GOLD (as in other observational data sources^29–31^). We could therefore not evaluate, who fulfilled the official criteria for a CKD diagnosis. Thus, not all pregnancies with eGFR<90 in this study population may formally fulfill the diagnostic criteria of CKD. However, only including pregnancies among patients with a recorded diagnosis for CKD would have introduced substantially larger bias, because under-recording of CKD diagnoses in CPRD GOLD is a known problem.^32^ To minimize the risk of including patients with CKD into our group of pregnancies with normal or high eGFR, we excluded pregnancies where any renal disease was recorded at any time before delivery. Second, we defined baseline eGFR based on SCr levels measured during baseline/trimester 1 because some patients do not obtain blood work until their pregnancy comes to medical attention (13.5% in our study). The GFR estimated during trimester 1 may have been slightly overestimated.^33^ However, fewer than 10% of pregnancies categorized as G2-high/G2-low or G3/G4 were selected based on SCr measurements in trimester 1. Excluding them would have introduced selection bias. Third, hypertension may increase the risk of prolonged increase in SCr levels after pregnancy,^34^ but we could not evaluate potential effect modification by hypertension due to the small sample size and could not distinguish whether hypertension was a cause or effect of reduced eGFR.^35^

# **References (section 2, 3, 4)**

1. Herrett E, Gallagher AM, Bhaskaran K, et al. Data Resource Profile: Clinical Practice Research Datalink (CPRD). *Int J Epidemiol*. 2015;44(3):827-836. doi:10.1093/ije/dyv098

2. Clinical Practice Research Datalink (CPRD). CPRD linked data. https://cprd.com/linked-data. Published 2021. Accessed November 30, 2021.

3. Hagberg Wilcox K, Robijn AL, Jick S. Maternal depression and antidepressant use during pregnancy and the risk of autism spectrum disorder in offspring. *Clin Epidemiol*. 2018;10:1599-1612. doi:10.2147/CLEP.S180618 LK

4. Van Rossum G, Drake FL. *Python 3 Reference Manual*. Scotts Valley, CA: CreateSpace; 2009.

5. Hunter JD. Matplotlib: A 2D graphics environment. 2007:90-95. doi:10.5281/zenodo.3714460

6. Centers for Disease Control and Prevention. Defining Adult Overweight & Obesity. https://www.cdc.gov/obesity/adult/defining.html. Published 2021. Accessed April 4, 2021.

7. Department of Health Renal Team. *The National Service Framework for Renal Services. Part One: Dialysis and Transplantation*. Vol 100.; 2004. http://www.ncbi.nlm.nih.gov/pubmed/14999827.

8. O’Sullivan JW, Stevens S, Hobbs FDR, et al. Temporal trends in use of tests in UK primary care, 2000-15: Retrospective analysis of 250 million tests. *BMJ*. 2018;363. doi:10.1136/bmj.k4666

9. Feakins B, Oke J, McFadden E, et al. Trends in kidney function testing in UK primary care since the introduction of the quality and outcomes framework: a retrospective cohort study using CPRD. *BMJ Open*. 2019;9(6):e028062. doi:10.1136/bmjopen-2018-028062

10. National Collaborating Centre for Chronic Conditions (UK). Chronic Kidney Disease: National Clinical Guideline for Early Identification and Management in Adults in Primary and Secondary Care. *London R Coll Physicians*. 2008;75(6):571.

11. Zhang JJ, Ma XX, Hao L, Liu LJ, Lv JC, Zhang H. A systematic review and meta-analysis of outcomes of pregnancy in CKD and CKD outcomes in pregnancy. *Clin J Am Soc Nephrol*. 2015;10(11):1964-1978. doi:10.2215/CJN.09250914

12. Wiles KS, Nelson-Piercy C, Bramham K. Reproductive health and pregnancy in women with chronic kidney disease. *Nat Rev Nephrol*. 2018;14(3):165-184. doi:10.1038/nrneph.2017.187

13. Kendrick J, Sharma S, Holmen J, Palit S, Nuccio E CM. Kidney Disease and Maternal and Fetal Outcomes in Pregnancy. *Am J Kidney Dis*. 2015;25(3):289-313. doi:110.1016/j.bbi.2017.04.008

14. Fischer MJ, Lehnerz SD, Hebert JR, Parikh CR. Kidney Disease Is an Independent Risk Factor for Adverse Fetal and Maternal Outcomes in Pregnancy. *Am J Kidney Dis*. 2004;43(3):415-423. doi:10.1053/j.ajkd.2003.10.041

15. Williams D, Davison J. Chronic kidney disease in pregnancy. *BMJ*. 2008;336(7637):211-215. doi:10.1136/bmj.39406.652986.BE

16. Munkhaugen J, Lydersen S, Romundstad PR, Widerøe TE, Vikse BE, Hallan S. Kidney function and future risk for adverse pregnancy outcomes: A population-based study from HUNT II, Norway. *Nephrol Dial Transplant*. 2009;24(12):3744-3750. doi:10.1093/ndt/gfp320

17. Diabetes UK. Diabetes in the UK 2010: Key statistics on diabetes. http://www.diabetes.org.uk/Documents/Reports/Diabetes_in_the_UK_2010.pdf. Published 2010. Accessed August 12, 2021.

18. Cabiddu G, Castellino S, Gernone G, et al. A best practice position statement on pregnancy in chronic kidney disease: the Italian Study Group on Kidney and Pregnancy. *J Nephrol*. 2016;29(3):277-303. doi:10.1007/s40620-016-0285-6

19. Wetmore JB, Guo H, Liu J, Collins AJ, Gilbertson DT. The incidence, prevalence, and outcomes of glomerulonephritis derived from a large retrospective analysis. *Kidney Int*. 2016;90(4):853-860. doi:10.1016/j.kint.2016.04.026

20. Bili E, Tsolakidis D, Stangou S, Tarlatzis B. Pregnancy management and outcome in women with chronic kidney disease. *Hippokratia*. 2013;17(2):163-168. http://www.ncbi.nlm.nih.gov/pubmed/24376324%0Ahttp://www.pubmedcentral.nih.gov/articlerender.fcgi?artid=PMC3743623.

21. United States Renal Data System. 2020 USRDS Annual Data Report: Epidemiology of kidney disease in the United States. *Natl Institutes Heal Natl Inst Diabetes Dig Kidney Dis Bethesda, MD*. 2020.

22. Cunningham FG, Cox SM, Harstad TW, Mason RA, Pritchard JA. Chronic renal disease and pregnancy outcome. *Am J Obstet Gynecol*. 1990;163(2):453-459. doi:10.1016/0002-9378(90)91175-C

23. Hou SH, Grossman SD, Madias NE. Pregnancy in women with renal disease and moderate renal insufficiency. *Am J Med*. 1985;78(2):185-194. doi:10.1016/0002-9343(85)90425-5

24. Imbasciati E, Gregorini G, Cabiddu G, et al. Pregnancy in CKD Stages 3 to 5: Fetal and Maternal Outcomes. *Am J Kidney Dis*. 2007;49(6):753-762. doi:10.1053/j.ajkd.2007.03.022

25. Wiles K, Webster P, Seed PT, et al. The impact of chronic kidney disease Stages 3–5 on pregnancy outcomes. *Nephrol Dial Transplant*. 2020:1-10. doi:10.1093/ndt/gfaa247

26. Piccoli GB, Fassio F, Attini R, et al. Pregnancy in CKD: Whom should we follow and why? *Nephrol Dial Transplant*. 2012;27(SUPPL. 3). doi:10.1093/ndt/gfs302

27. Piccoli GB, Vigotti FN, Cabiddu G, et al. Risk of adverse pregnancy outcomes in women with CKD. *J Am Soc Nephrol*. 2015;26(8):2011-2022. doi:10.1681/ASN.2014050459

28. Office for National Statistics. Birth characteristics in England and Wales: 2019. Statistical bulletin. https://www.ons.gov.uk/peoplepopulationandcommunity/birthsdeathsandmarriages/livebirths/bulletins/birthcharacteristicsinenglandandwales/latest#age-of-parents. Published 2020. Accessed July 20, 2021.

29. Vestergaard SV, Christiansen CF, Thomsen RW, Birn H, Heide-Jørgensen U. Identification of patients with ckd in medical databases a comparison of different algorithms. *Clin J Am Soc Nephrol*. 2021;16(4):543-551. doi:10.2215/CJN.15691020

30. Vrijlandt WAL, de Jong MFC, Prins JR, et al. Prevalence of chronic kidney disease in women of reproductive age and observed birth rates. *J Nephrol*. 2023;(0123456789). doi:10.1007/s40620-022-01546-z

31. Hayes JF, Osborn DPJ, Francis E, et al. Prediction of individuals at high risk of chronic kidney disease during treatment with lithium for bipolar disorder. *BMC Med*. 2021;19(1):99. doi:10.1186/s12916-021-01964-z

32. de Lusignan S, Chan T, Stevens P, et al. Identifying patients with chronic kidney disease from general practice computer records. *Fam Pract*. 2005;22(3):234-241. doi:10.1093/fampra/cmi026

33. Cheung KL, Lafayette RA. Renal Physiology of Pregnancy. *Adv Chronic Kidney Dis*. 2013;20(3):209-214. doi:10.1053/j.ackd.2013.01.012

34. Ku E, Lee BJ, Wei J, Weir MR. Hypertension in CKD: Core Curriculum 2019. *Am J Kidney Dis*. 2019;74(1):120-131. doi:10.1053/j.ajkd.2018.12.044

35. Muntner P, Anderson A, Charleston J, et al. Hypertension Awareness, Treatment, and Control in Adults With CKD: Results From the Chronic Renal Insufficiency Cohort (CRIC) Study. *Am J Kidney Dis*. 2010;55(3):441-451. doi:10.1053/j.ajkd.2009.09.014

1. Office for National Statistics. Birth characteristics in England and Wales: 2019. Statistical Bulletin. Published 2019. Accessed July 15, 2021. https://www.ons.gov.uk/peoplepopulationandcommunity/birthsdeathsandmarriages/livebirths/bulletins/birthcharacteristicsinenglandandwales/2017 [↑](#footnote-ref-1)
2. Clinical GAB ≥37 weeks [↑](#footnote-ref-2)
3. Clinical GAB <37 weeks [↑](#footnote-ref-3)
4. Clinical GAB ≥37 weeks [↑](#footnote-ref-4)
5. Clinical GAB <37 weeks [↑](#footnote-ref-5)
